# Supplementary material for: Fracture toughness of mixed-mode anticracks in highly porous materials
Source: Nat Commun. 2024 Sep 2;15:7379. doi: 10.1038/s41467-024-51491-7 (PMC11368921; doi:10.1038/s41467-024-51491-7)
Supplement: Supplementary file 1 — Supplementary Information [file 41467_2024_51491_MOESM1_ESM.pdf]

Supplementary information: fracture toughness of mixed-mode anticracks in highly porous materials

Valentin Adam[1,2], Bastian Bergfeld[2], Philipp Weißgraeber[3], Alec van Herwijnen[2] and Philipp L. Rosendahl[1*]

[1]Institute of Structural Mechanics and Design, Department of Civil and Environmental Engineering, Technical University of Darmstadt, Franziska-Braun-Str. 3, 64285 Darmstadt, Germany.
[2]WSL Institute for Snow and Avalanche Research SLF, Flüelastr. 11, 7260 Davos, Switzerland.
[3]Chair of Lightweight Design, Faculty of Mechanical Engineering and Marine Technology, University of Rostock, Albert-Einstein-Straße 2, 18059 Rostock, Germany.

*Corresponding author(s). E-mail(s): rosendahl@ismd.tu-darmstadt.de;

Supplementary methods

Field site and snowpack

Our experiments were performed between February 18 and March 10, 2022 on a flat and uniform site in Flüela valley near Davos, Switzerland at an altitude of 1640 m (Fig. S1). The site itself was on the roof of two buildings in a forest opening protected from wind. Most experiments were performed on the roof of building A (Fig. 3 in the main text), and after it was cleared from snow, we also carried out experiments on building B (Fig. 3 in the main text). The presence of a nearby creek, the absence of direct sunlight in winter, and the cold concrete roof (typically below 0 °C), created favorable conditions for the formation and preservation of surface hoar. The weak layer tested consisted of surface hoar, buried by a snowfall at the beginning of January 2022, with an average weak layer thickness of 9.02 mm.

We characterized the snowpack using manual snow profiles[1] (Fig. 4 in the main text) Microstructure and density of the weak layer were analyzed using computer-tomography (Fig. S2). The properties of the layered slab were characterized using density measurements. For this purpose, we used a cylindrical density cutter with 50 cm^3 volume and 23 mm inner diameter (Fig. S3b). Each 120 mm thick slab was resolved in with four density measurements (Fig. S3a), accounting for temporal evolution of density-dependent slab properties. For our calculations, we used the arithmetic mean density per layer per experimental day (Fig. S3a).

For successful experiments, specific snowpack properties were necessary, in particular surrounding the weak layer. The substratum needed a particular level of cohesion or bonding strength, usually found in small grains with a density exceeding 250 kg/m^3, to support the snow block during tilting. Similarly, the slab had to be easily profiled while also supporting additional weights. We met these conditions with dense layers of rounded grains both above and right below the weak layer of interest between mid February and mid March of 2022 (Fig. 3 in the main text).

Experimental procedure

We designed our mixed-mode mixed-mode fracture tests (MMFTs) by adapting the propagation saw test[2–4] (PST) to enable testing under variable slope inclinations. To extract snow columns from the snowpack, we employed a U-shaped aluminum sled (3 mm thick with 60 mm flanges, 300 mm width, and 1000 mm

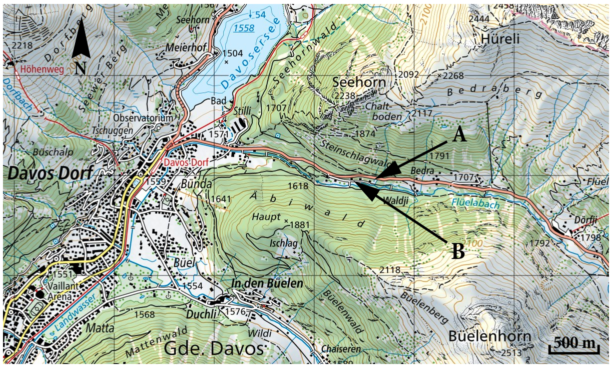

Supplementary Figure S1 | Geographic location of the field site. Topographical map (https://map.geo.admin.ch, accessed Jan 29, 2024) of the location of the two field sites A and B (WGS 84, 46.80773° N, 9.86999° E). Federal Office of Topography ©swisstopo.

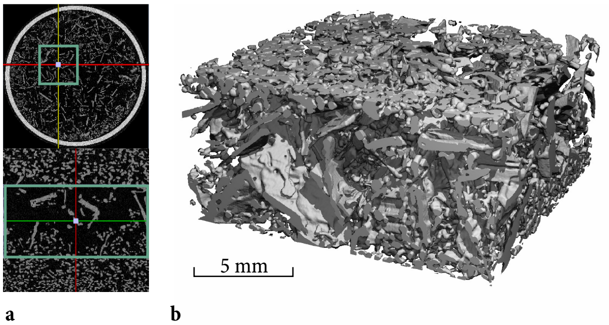

Supplementary Figure S2 | Computer-tomography scan of the weak layer. **a** Horizontal (top) and vertical (bottom) cross sections with boxed volume of interest (VOI) of the surface-hoar weak-layer sample extracted on March 7, 2022 at site A. **b** Rendering of the recorded VOI with a volume fraction of ice of 0.19 corresponding to a density of $\rho_{\mathrm{wl}} = 174\,\mathrm{kg/m^3}$.

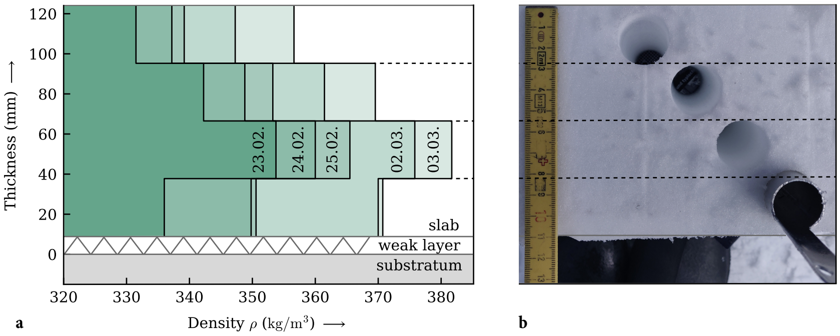

Supplementary Figure S3 | Slab density. a Evolution of the slab density on site A over a period of 9 days. Layerwise mean densities per day are shown. The mean density of the substratum was $\rho_\mathrm{b} = 339\,\mathrm{kg/m^3}$. **b** Location of four density measurements through the thickness of each 120 mm slab using a $50\,\mathrm{cm^3}$ density cutter.

length) (Fig. S4a). Utilizing a spirit level to maintain horizontal alignment, we inserted the sled into the snowpack's sidewall, ensuring that its flanges rested directly beneath the weak layer. This facilitated the isolation of a snow column with a 60 mm thick base layer (substratum). We cut around the sled on all sides using a 1 m snow saw to sever the snow block specimen (Fig. S4b). After reducing the slab's thickness to 150 mm, we applied a custom-made profiling device, creating serrated cuts on its top surface. This resulted in a mean slab thickness from the weak layer to the base of the serrated cuts of 115 mm. Guided by side rails, the sled–snow block assembly was lifted onto the tilting rig (Fig. S4c). Wood screws, penetrating the substratum through circular holes in the aluminum sled, prevented sliding of the snow block even at elevated inclinations (Fig. S4c).

The tilting device comprised a base plate, which was pivoted on a metal foot on one end and suspended on a steel cable from a tower made of scaffolding poles on the other. This configuration enabled the assembly to be tilted between 0° and 65° (Fig. S4d). The titled snow block was loaded with 12 variable weights distributed into notches (Fig. S4e). Each weight consisted of a rectangular hollow steel profile (500 mm length) with up to three metal rods (600 mm length). The profile–rod assemblies weighed up to 1 kg each, enabling the application of different load levels without altering the slab bending stiffness. In certain instances, an additional row of weights was added for very high surface loading. The titling angle was measured using an analog inclinometer aligned with the weak layer. To initiate the fracture process, we introduced a cut into the weak layer by pushing the unserrated back of a 2 mm thick snow saw (450 mm length, 60 mm width) into the weak layer (Fig. S4e). Two operators from both sides ensured that the saw remained within the weak layer. We cut at a constant travel speed of approximately 70 mm/s (Fig. S4e). The cutting speed employed here is typical for conducting propagation saw tests. Note that variations in cutting speed between 8 mm/s and 260 mm/s do not significantly affect the critical cut length.[5] When the artificially induced weak-layer crack became unstable and propagated through the entire sample, the critical cut length from saw tip to slab face was measured on both sidewalls and averaged when the cut was not perfectly perpendicular (Fig. S4f).

In total, we conducted 102 MMFTs and obtained 88 valid results. Experiments were discarded when we encountered increased resistance while cutting, indicating that the cut did not remain in the weak layer. Experiments with cut lengths exceeding 50 cm were also excluded due to the limited effective length of the slab, which was only 100 cm.

Data fitting procedure

The interaction laws examined in this work are two-dimensional implicit nonlinear models

$$0 \approx r(\boldsymbol{x}_i;\ \boldsymbol{\beta}), \tag{S1}$$

where $\boldsymbol{\beta} = (\mathcal{G}_\mathrm{Ic}, \mathcal{G}_\mathrm{IIc}, n, m)^\intercal$ is the vector of model parameters and $\boldsymbol{x}_i = (\mathcal{G}_\mathrm{I}, \mathcal{G}_\mathrm{II})_i^\intercal$ is the vector of independent variables, i.e., the vector of $i = 1, \ldots, |S|$ observations (see Eq. (1) of the main text). Owing to measurement errors in the observations $\boldsymbol{x}_i$, the model $r(\boldsymbol{x}_i;\ \boldsymbol{\beta})$ can only approximate 0. Because of the implicit relationship and because of uncertainties in the independent variables, the parameters $\boldsymbol{\beta}$ were estimated using a weighted orthogonal-distance-regression procedure.[6–8] Accounting for the measurement errors, the models satisfy

$$0 = r(\boldsymbol{x}_i + \boldsymbol{\delta_i};\ \boldsymbol{\beta}), \quad \text{for} \quad i = 1, \ldots, |S|, \tag{S2}$$

where $\boldsymbol{\delta} \in \mathbb{R}^2$ is the vector of unknown errors. The implicit orthogonal-distance-regression problem is finding the $\boldsymbol{\beta}$ for which the sum of the squares of the $|S|$ orthogonal distances from the curve $r(\boldsymbol{x}, \boldsymbol{\beta})$ to the $|S|$ data points is minimized. This is expressed by the optimization problem

$$\min_{\boldsymbol{\beta},\boldsymbol{\delta}} \sum_{i=1}^{N} \boldsymbol{\delta}_i^\intercal \boldsymbol{W}_i\, \boldsymbol{\delta}_i, \tag{S3}$$

subject to

$$0 = r(\boldsymbol{x}_i + \boldsymbol{\delta_i};\ \boldsymbol{\beta}), \tag{S4}$$

where the diagonal matrix

$$\boldsymbol{W} = \begin{pmatrix} \sigma_\mathrm{I}^{-2} & 0 \\ 0 & \sigma_\mathrm{II}^{-2} \end{pmatrix}, \tag{S5}$$

accounts for unequal error variances σ_I^2 and σ_II^2 in $\mathcal{G}_\mathrm{I}$ and $\mathcal{G}_\mathrm{II}$, respectively. The optimization problem was solved using a trust-region Levenberg–Marquardt procedure.[8] Jacobian matrices with respect to parameters and independent variables were computed explicitly and supplied to the algorithm. The goodness of fit is assessed using the residual variance expressed by

$$\chi_\nu^2 = \frac{1}{\nu} \sum_{i=1}^{N} \boldsymbol{\delta}_i^\intercal \boldsymbol{W}_i\, \boldsymbol{\delta}_i, \tag{S6}$$

where $\nu = N - P$ is the number of degrees of freedom obtained from the number of observations with nonzero weight N and the number of estimated model parameters P. A χ_ν^2 value close to 1

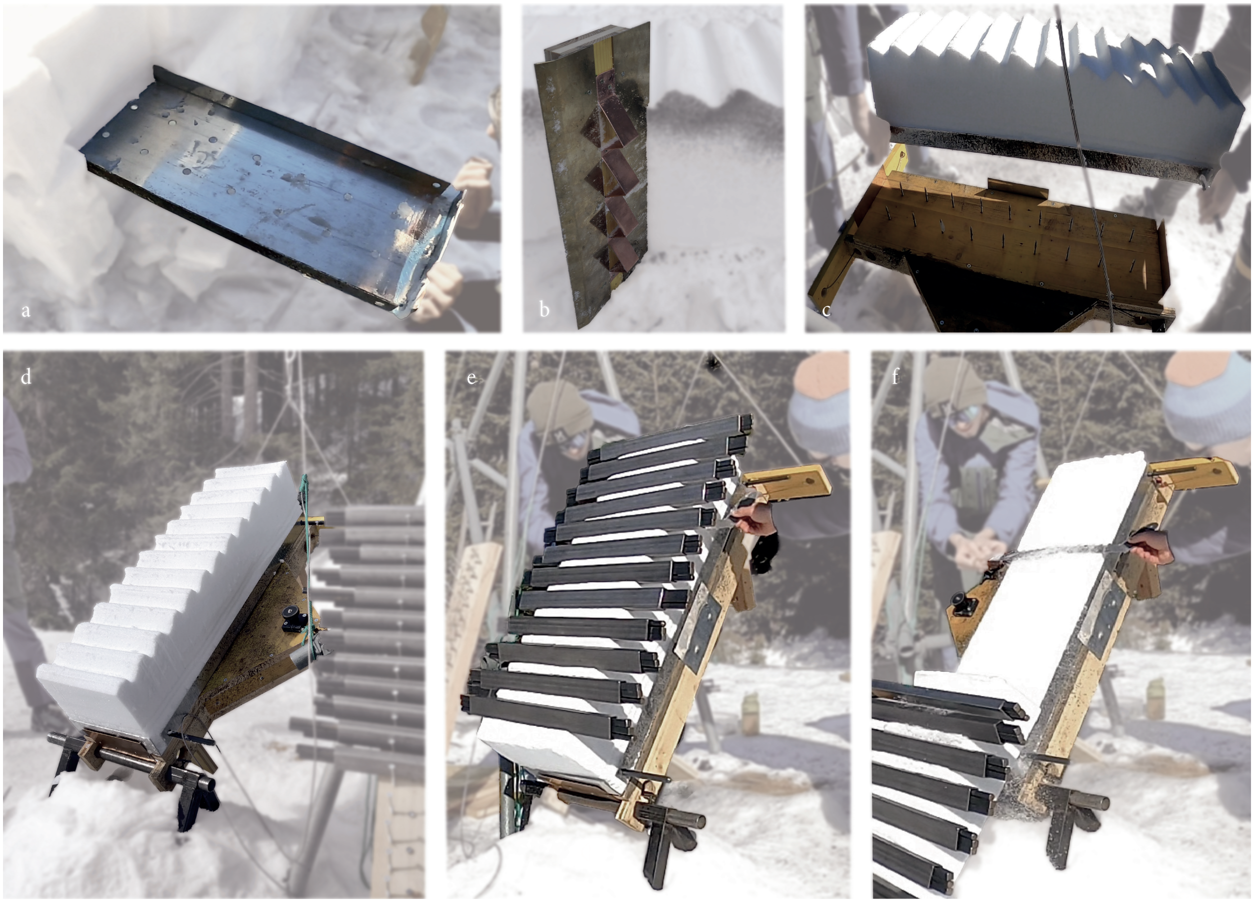

Supplementary Figure S4 | Experimental procedure. a Aluminum sled pushed into the snowpack to facilitate the extraction of the snow column of interest. **b** Profiling device used to serrate the slab's top surface to support the addition of weights. **c** Placement of snowpack–sled assembly onto the tilting rig with guiding plates on the sides and the bottom edge to ensure alignment. Screws punch into the substratum to prevent sliding. **d** Assembly tilted to final inclination before addition of weights. **e** Weak-layer cut introduced with the back of a snow saw by two people ensuring parallel movement through the weak layer. **f** Slab sliding after unstable propagation of the introduced crack. The cut length is recorded from the end of the saw to the end of the slab.

generally indicates that the model fits the data well, where < 1 indicates overfitting and > 1 underfitting. For datasets with intrinsic scatter beyond measurement errors—such as snow science and fracture mechanics where individual variation is significant—the model is not expected to predict each point accurately. In these cases, a higher χ^2_ν value can be acceptable if residuals are randomly distributed and do not exhibit patterns.

Interaction-law identification

Fracture-toughness interaction laws have been proposed and examined by many authors.[9] In the following, we illustrate their differences and justify our choice of a power-law interaction model using two characteristic examples.

Compare the best fit of the power-law interaction model given in Eq. (1) of the main text (Fig. 2)

$$0 \approx r_1(\boldsymbol{x}_i;\ \boldsymbol{\beta}_1) \equiv \left(\frac{\mathcal{G}_\mathrm{I}^i}{\mathcal{G}_\mathrm{Ic}}\right)^{\frac{1}{n}} + \left(\frac{\mathcal{G}_\mathrm{II}^i}{\mathcal{G}_\mathrm{IIc}}\right)^{\frac{1}{m}} - 1, \tag{S7}$$

where $\boldsymbol{\beta}_1 = (\mathcal{G}_\mathrm{Ic}, \mathcal{G}_\mathrm{IIc}, n, m)^\intercal$, to the mixed-mode interaction law proposed by Benzeggagh and Kenane[10]

$$0 \approx r_2(\boldsymbol{x}_i;\ \boldsymbol{\beta}_2) \equiv \frac{\mathcal{G}_\mathrm{Ic} + (\mathcal{G}_\mathrm{IIc} - \mathcal{G}_\mathrm{Ic})\,\psi_i^k}{\mathcal{G}^i + \mathcal{G}_\mathrm{II}^i} - 1, \tag{S8}$$

where

$$\psi_i = \frac{\mathcal{G}_\mathrm{II}^i}{\mathcal{G}_\mathrm{I}^i + \mathcal{G}_\mathrm{II}^i}, \tag{S9}$$

is the mode ratio and $\boldsymbol{\beta}_2 = (\mathcal{G}_\mathrm{Ic}, \mathcal{G}_\mathrm{IIc}, k)^\intercal$ the vector of model parameters (Fig. S5). Equation (S8) was proposed to capture mixed-mode fracture toughness under tension–shear interaction,[10] where the total energy release rate $\mathcal{G} = \mathcal{G}_\mathrm{I} + \mathcal{G}_\mathrm{II}$ is observed as a monotonous function of the mode ratio, expressed in the ψ^k-term.[10,11] Our data show that for compression–shear interaction, the total energy release rate is not monotonous with respect to the mode ratio ψ (Figs. 2b and S5b). This incompatibility results in a linear relationship $\mathcal{G}(\psi)$, i.e., $k = 1.0$ (Table S1), as the best fit of Eq. (S8) and in a significantly larger residual variance χ^2_ν (4.16 vs. 3.14, Figs. 2b and S5b). Many other interaction laws proposed in literature[9,12]—for dense isotropic materials or fiber-reinforced plastics under combined tension and shear—are of similar characteristics as Eq. (S8). However, as they are aimed at both other classes of materials and other load interactions, they did not provide a better fit to our data.

While the mode I fracture toughness estimates of both models are similar, their mode II representations are much different (Tables 1 and S1). Owing to their exceptionally low density, we assume that the tensile mode I fracture toughness of highly

Supplementary Table S1 | Best fit parameters. Interaction-law parameters of Eq. (S8) identified from a weighted orthogonal distance regression.

Weak layer type	$\mathcal{G}_{\mathrm{Ic}}$ (J/m^2)	$\mathcal{G}_{\mathrm{IIc}}$ (J/m^2)	k
Surface hoar	0.59 ± 0.03	1.12 ± 0.07	1.0

porous weak layers is very small. That is, we expect a mixed-mode law that captures the interaction of mode II with both mode I compression and mode I tension to decrease sharply on the tension side. For this reason, we expect a vanishing or small but positive gradient $\partial\mathcal{G}_{\mathrm{II}}/\partial\mathcal{G}_{\mathrm{I}}$ for $\mathcal{G}_{\mathrm{I}} \to 0$ (Figs. 2a). However, for the best fit of Eq. (S8), we observe a steep, negative gradient (Fig. S5a).

Eq. (S8) was proposed to account for tension–shear interaction. We observe that the mechanics of compression–shear interaction are quite different and that interactions laws are not directly transferable.

Model derivation

The mechanical model used in this work results from a series of articles in which different components of the theory have been derived and tested. The novelty, here, is the treatment of added surface loads. To help readers who would not be familiar already with this literature, we provide a summary how physical components have been validated. In the following, we refer to the works of Weißgraeber & Rosendahl.[13–15]

Governing equations. We model a stratified snow cover as a system comprised of i) a snow slab, represented by an arbitrarily layered beam, that rests ii) on a weak layer, represented by an elastic foundation. The beam kinematics and its constitutive behavior are derived from first-order shear deformation theory of laminated plates under cylindrical bending.[16] The weak layer is modeled as a so-called weak interface.[17] The concept simplifies the kinematics of the weak layer and allows for efficient analyses of interface configurations that exhibit a strong elastic contrast. The weak interface can be understood as an infinite set of smeared springs with normal and shear stiffness attached to the bottom side of the slab. Weak interface models are common for the analysis of cracks in thin, compliant layers.[18–20] The analysis of this system yields fully coupled bending, extension and shear deformations of both slab and weak layer.

Consider the segment of the stratified snow pack on an inclined slope of angle φ shown in Fig. S6. As typical for beam analyses, the axial coordinate x points left-to-right along the beam midplane and is zero at its left end. The thickness coordinate z is perpendicular to the midplane, points downwards and is zero at the center line. Slope angles φ are counted positive about the y axis of the right-handed Cartesian coordinate system (counterclockwise). Note that on inclined slopes ($\varphi \neq 0$), the axial and normal beam axes (x and z) do not coincide with the horizontal and vertical directions.

The slab with total thickness h is composed of N layers with individual ply thicknesses $h_i = z_{i+1} - z_i$, each assumed homogeneous and isotropic (Fig. S7). Young's modulus, Poisson's ratio and density of each layer are denoted by E_i, ν_i and ρ_i, respectively. The weak layer of thickness t can be anisotropic and its normal and tangential stiffnesses are

$$k_{\mathrm{n}} = \frac{E'_{\mathrm{wl}}}{t}, \tag{S10a}$$

where $E'_{\mathrm{wl}} = E_{\mathrm{wl}}/(1-\nu^2)$ is the weak layer's plane-strain elastic modulus and

$$k_{\mathrm{t}} = \frac{G_{\mathrm{wl}}}{t}, \tag{S10b}$$

where G_{wl} is the weak layer's plane-strain shear modulus, respectively. To account for anisotropic weak layers, these constants can be defined from independent stiffness properties. It is to note, that since the weak layer is connected to the slab, an intrinsic coupling of shear and normal deformation of the weak layer occurs even when the stiffnesses k_{n} and k_{t} are defined independently.

The slab is loaded by its own weight, i.e., the gravitational load q, and an external load F (e.g., a skier or added weights) in vertical direction. The gravity load corresponds to the sum of the weight of all layers

$$q = g\sum_{i=1}^{N} h_i\rho_i. \tag{S11}$$

It is split into a normal component $q_{\mathrm{n}} = q\cos\varphi$ and a tangential component $q_{\mathrm{t}} = -q\sin\varphi$ that are introduced as line loads. The tangential gravity line load acts at center of gravity in thickness direction

$$z_{\mathrm{s}} = \frac{\sum_{i=1}^{N}(z_i + z_{i+1})h_i\rho_i}{2\sum_{i=1}^{N} h_i\rho_i}, \tag{S12}$$

in the slab, where $(z_i + z_{i+1})/2$ yields each layer's center z-coordinate. For relevant slab thicknesses the external load can be modeled as a point load and is introduced as a force with a normal component $F_{\mathrm{n}} = F\cos\varphi$ and a tangential component $F_{\mathrm{t}} = -F\sin\varphi$.

Deformations of the slab are described by means of the first-order shear deformation theory (FSDT) of laminated plates under cylindrical bending.[16] By dropping the Kirchhoff assumption of orthogonality of cross sections and midplane, this allows for the consideration of shear deformations. We consider midplane deflections w_0, midplane tangential displacements u_0 and the rotation ψ of cross sections. The quantities define the displacement field of the beam according to

$$w(x,z) = w_0(x), \tag{S13a}$$
$$u(x,z) = u_0(x) + z\psi(x). \tag{S13b}$$

At the interface between slab and weak layer ($z = h/2$), the displacement fields of slab (u, w) and weak-layer (v, ω) coincide. Using Eqs. (S13a) and (S13b), this yields $\bar{v} = \bar{u} = u_0 + \psi\, h/2$ and $\bar{\omega} = \bar{w} = w_0$, where the bar indicates quantities at the interface. Modeling the weak layer as an elastic foundation of an infinite set of smeared linear elastic springs, yields constant strains and consequently a constant deformation gradient through its thickness. Hence, weak-layer stresses can be expressed through the differential deformation between the lower boundary of the weak layer ($v = \omega = 0$) and its deformations at the interface:

$$\begin{aligned}\sigma_{zz}(x) &= E_{\mathrm{wl}}\varepsilon_{zz}(x) = E_{\mathrm{wl}}\frac{\mathrm{d}\omega(x,z)}{\mathrm{d}z} = E_{\mathrm{wl}}\frac{0-\bar{\omega}(x)}{t} \\ &= -k_{\mathrm{n}}w_0(x),\end{aligned} \tag{S14a}$$

$$\begin{aligned}\tau_{xz}(x) &= G_{\mathrm{wl}}\gamma_{xz}(x) = G_{\mathrm{wl}}\left(\frac{\mathrm{d}v(x,z)}{\mathrm{d}z} + \frac{\mathrm{d}\omega(x,z)}{\mathrm{d}x}\right) \\ &= G_{\mathrm{wl}}\left(\frac{0-\bar{v}(x)}{t} + \frac{\bar{\omega}'(x)}{2}\right) \\ &= k_{\mathrm{t}}\left(\frac{t}{2}w_0'(x) - u_0(x) - \frac{h}{2}\psi(x)\right).\end{aligned} \tag{S14b}$$

From the free body-cut of an infinitesimal beam section of the layered slab (Fig. S8), we obtain the equilibrium conditions of the section forces and moments:

$$0 = \frac{\mathrm{d}N(x)}{\mathrm{d}x} + \tau(x) + q_{\mathrm{t}} + p_{\mathrm{t}}, \tag{S15a}$$

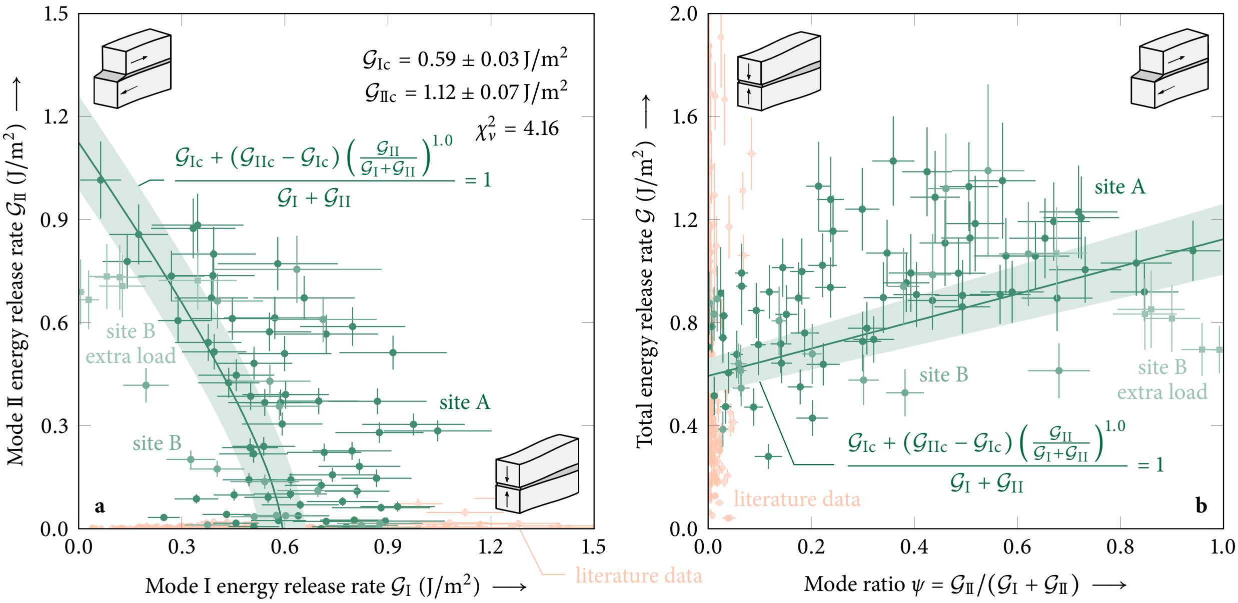

Supplementary Figure S5 | Best fit of tension–shear mixed-mode interaction law. a Mode I/II composition of critical energy release rates at the onset of unstable crack propagation from this work ($N = 88$, green) and literature[5] ($N = 183$, orange) with best fit ($p < 0.001$) of Eq. (S8). **b** Total energy release rate $\mathcal{G} = \mathcal{G}_{\mathrm{I}} + \mathcal{G}_{\mathrm{II}}$ as a function of mode ratio ψ (mode II fraction). A monotonous model of the total energy release rate vs. mode ratio ψ, e.g., Eq. (S8), cannot capture the local maximum evident in the data.

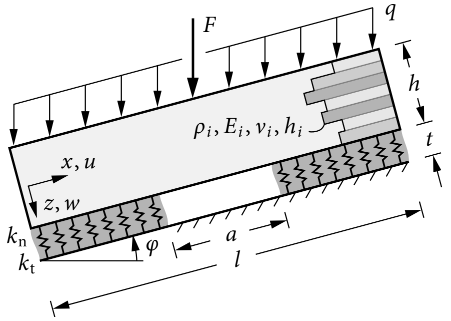

Supplementary Figure S6 | Mechanical model. Stratified snowpack composed of an arbitrary number of slab layers and a weak layer modeled as an elastic foundation.

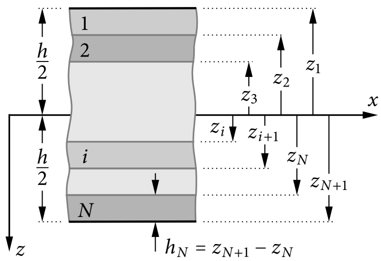

Supplementary Figure S7 | Slab layering. Slab of total thickness h composed of N individual layers. A layer i is characterized by its height h_i and its the top and bottom coordinates z_i and z_{i+1}, respectively.

$$0 = \frac{\mathrm{d}V(x)}{\mathrm{d}x} + \sigma(x) + q_{\mathrm{n}} + p_{\mathrm{n}}, \tag{S15b}$$

$$0 = \frac{\mathrm{d}M(x)}{\mathrm{d}x} - V(x) + \frac{h+t}{2}\tau(x) + z_{\mathrm{s}} q_{\mathrm{t}} - \frac{h}{2} p_{\mathrm{t}}. \tag{S15c}$$

Note the addition of normal and tangential surface loads p_{n} and p_{t}, respectively.[15] To connect the slab section forces (normal force N, shear force V, and bending moment M) to the deformations of the layered slab, we make use of the mechanics of composite laminates. First-order shear deformation theory of laminate plates under cylindrical bending yields

$$\begin{pmatrix} N(x) \\ M(x) \end{pmatrix} = \begin{pmatrix} A_{11} & B_{11} \\ B_{11} & D_{11} \end{pmatrix} \begin{pmatrix} u_0'(x) \\ \psi'(x) \end{pmatrix}, \tag{S16a}$$

and

$$V(x) = \kappa A_{55} \left(w_0'(x) + \psi(x) \right). \tag{S16b}$$

These constitutive equations contain the extensional stiffness A_{11}, the bending stiffness D_{11}, the bending–extension coupling stiffness B_{11}, and the shear stiffness κA_{55} of the layered slab. The coupling stiffness B_{11} accounts for the bending–extension coupling of asymmetrically layered systems such as bimetal bars. These stiffness quantities are obtained by weighted[1] integration of the individual ply stiffness properties:

$$A_{11} = \int_{-h/2}^{h/2} \frac{E(z)}{1-\nu(z)^2}\,\mathrm{d}z = \sum_{i=1}^{N} \frac{E_i}{1-\nu_i^2} h_i, \tag{S17a}$$

$$B_{11} = \int_{-h/2}^{h/2} \frac{E(z)}{1-\nu(z)^2} z\,\mathrm{d}z = \frac{1}{2} \sum_{i=1}^{N} \frac{E_i}{1-\nu_i^2} \left(z_{i+1}^2 - z_i^2 \right), \tag{S17b}$$

$$D_{11} = \int_{-h/2}^{h/2} \frac{E(z)}{1-\nu(z)^2} z^2\,\mathrm{d}z = \frac{1}{3} \sum_{i=1}^{N} \frac{E_i}{1-\nu_i^2} \left(z_{i+1}^3 - z_i^3 \right), \tag{S17c}$$

[1]Weighted by the moment of area of the cross-section of zeroth, first, and second order.

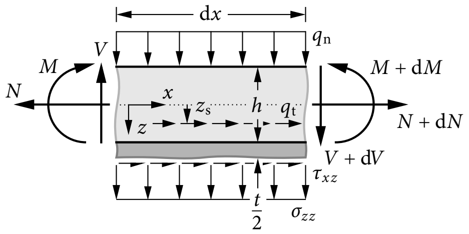

Supplementary Figure S8 | Equilibrium conditions. Free-body cut of an infinitesimal segment of length of the layered slab of height with half of the weak layer.

$$A_{55} = \int_{-h/2}^{h/2} G(z)\,\mathrm{d}z = \sum_{i=1}^{N} G_i h_i. \tag{S17d}$$

The shear correction factor κ complements the shear stiffness κA_{55}. It is set to 5/6 as a good approximation for the layered slab of rectangular cross-section.[21] The above quantities are given for the case of isotropic layers. Orthotropic layers can be considered following the same approach by using directional elastic properties of the individual layers instead of an isotropic Young's modulus.

In the special case of a homogeneous, isotropic slab with Young's modulus E_{sl} and Poisson's ratio ν, the laminate stiffnesses take the homogeneous stiffness properties well-known from beam theory:

$$A_{11} = \frac{E_{\mathrm{sl}} h}{1-\nu^2}, \tag{S18a}$$

$$D_{11} = \frac{E_{\mathrm{sl}} h^3}{12\left(1-\nu^2\right)}, \tag{S18b}$$

$$A_{55} = \frac{E_{\mathrm{sl}} h}{2\,(1+\nu)}, \tag{S18c}$$

and the coupling stiffness vanishes ($B_{11} = 0$).

System of differential equations. The equations of the kinematics of the weak layer, (S14a) and (S14b), the equilibrium conditions, (S15a) to (S15c), and the constitutive equations of the layered beam with first-order shear deformation theory, (S16a) and (S16b), provide a complete description of the mechanics of the layered snowpack and constitute a system of ordinary differential equations (ODEs) of second order.

With the first derivative of the constitutive equation of the normal force (S16a)′ inserted into the equilibrium of horizontal forces (S15a), we obtain

$$0 = A_{11} u_0''(x) + B_{11} \psi_0''(x) + \tau(x) + q_{\mathrm{t}}. \tag{S19}$$

Likewise, with the first derivative of the constitutive equation of the shear force (S16b)′ and the vertical force equilibrium (S15b), we have:

$$0 = \kappa A_{55}(w_0''(x) + \psi'(x)) + \sigma(x) + q_{\mathrm{n}}. \tag{S20}$$

The first derivative of the constitutive equation of the bending moment (S16a)′ with the balance of moments (S15c), yields

$$\begin{aligned} 0 &= B_{11} u_0''(x) + D_{11} \psi''(x) - \kappa A_{55}\left(w_0'(x) + \psi(x)\right) \\ &\quad + \frac{h+t}{2}\tau(x) + z_{\mathrm{s}} q_{\mathrm{t}}. \end{aligned} \tag{S21}$$

We then insert the definition of the shear stresses (S14b) into Eq. (S19) to obtain

$$\begin{aligned} 0 &= A_{11} u_0''(x) - k_{\mathrm{t}} u_0(x) - k_{\mathrm{t}} \frac{t}{2} w_0'(x) \\ &\quad + B_{11}\psi''(x) - k_{\mathrm{t}} \frac{h}{2}\psi(x) + q_{\mathrm{t}}. \end{aligned} \tag{S22}$$

Inserting the normal stress definition (S14a) into Eq. (S20), yields

$$0 = \kappa A_{55} w_0''(x) - k_{\mathrm{n}} w_0(x) + \kappa A_{55}\psi'(x) + q_{\mathrm{n}}, \tag{S23}$$

and, again, inserting the shear stress (S14b) into Eq. (S21), yields

$$\begin{aligned} 0 &= B_{11} u_0''(x) - k_{\mathrm{t}} \frac{h+t}{2} u_0(x) + D_{11}\psi''(x) \\ &\quad + \left(\frac{h+t}{2}\frac{t}{2}k_{\mathrm{t}} - \kappa A_{55}\right) w_0'(x) \\ &\quad - \left(\kappa A_{55} + \frac{h+t}{2}\frac{h}{2}k_{\mathrm{t}}\right)\psi(x) + z_{\mathrm{s}} q_{\mathrm{t}}. \end{aligned} \tag{S24}$$

Equations (S22) to (S24) constitute a system of linear ordinary differential equations of second order with constant coefficients of the deformation variables $u(x)$, $w(x)$, $\psi(x)$ that describes the mechanical behavior of a layered beam on a weak layer.

Using the vector of unknown functions

$$\boldsymbol{z}(x) = \begin{bmatrix} u_0(x) & u_0'(x) & w_0(x) & w_0'(x) & \psi(x) & \psi'(x) \end{bmatrix}^{\top}, \tag{S25}$$

the ODE system can be written as a system of first-order for the form

$$\boldsymbol{A}\boldsymbol{z}'(x) + \boldsymbol{B}\boldsymbol{z}(x) + \boldsymbol{d} = 0, \tag{S26}$$

with the matrices

$$\boldsymbol{A} = \begin{bmatrix} 1 & 0 & 0 & 0 & 0 & 0 \\ 0 & A_{11} & 0 & 0 & 0 & B_{11} \\ 0 & 0 & 1 & 0 & 0 & 0 \\ 0 & 0 & 0 & \kappa A_{55} & 0 & 0 \\ 0 & 0 & 0 & 0 & 1 & 0 \\ 0 & B_{11} & 0 & 0 & 0 & D_{11} \end{bmatrix}, \tag{S27}$$

and

$$\boldsymbol{B} = \begin{bmatrix} 0 & -1 & 0 & 0 & 0 & 0 \\ -k_{\mathrm{t}} & 0 & 0 & k_{\mathrm{t}}\frac{t}{2} & -k_{\mathrm{t}}\frac{h}{2} & 0 \\ 0 & 0 & 0 & -1 & 0 & 0 \\ 0 & 0 & -k_{\mathrm{n}} & 0 & 0 & \kappa A_{55} \\ 0 & 0 & 0 & 0 & 0 & -1 \\ -\frac{h+t}{2}k_{\mathrm{t}} & 0 & 0 & B_{64} & B_{65} & 0 \end{bmatrix}, \tag{S28}$$

where

$$B_{64} = k_{\mathrm{t}}\frac{h+t}{4}t - \kappa A_{55}, \text{ and } B_{65} = -k_{\mathrm{t}}\frac{h+t}{4}h - \kappa A_{55},$$

and the vector

$$\boldsymbol{d} = \begin{bmatrix} 0 & q_{\mathrm{t}} + p_{\mathrm{t}} & 0 & q_{\mathrm{n}} + p_{\mathrm{n}} & 0 & z_{\mathrm{s}} q_{\mathrm{t}} - \frac{h}{2} p_{\mathrm{t}} \end{bmatrix}^{\top}. \tag{S29}$$

Note the addition of surface loads p_{n} and p_{t}.[15] The system (S26) can be rearranged into the form

$$\boldsymbol{z}'(x) = \boldsymbol{K}\boldsymbol{z}(x) + \boldsymbol{q}, \tag{S30}$$

where

$$\boldsymbol{K} = -\boldsymbol{A}^{-1}\boldsymbol{B}, \tag{S31a}$$

$$\boldsymbol{q} = -\boldsymbol{A}^{-1}\boldsymbol{d}. \tag{S31b}$$

The solution of the nonhomogeneous ODE system (S30) is composed of a complementary solution vector $\boldsymbol{z}_{\mathrm{h}}(x)$ and a particular integral vector $\boldsymbol{z}_{\mathrm{p}}$, where the latter is constant in the present case. The complementary solution can be obtained from an eigenanalysis of the system matrix $\boldsymbol{K}$. Depending on the layering and the material properties, $\boldsymbol{K}$ has six real or complex eigenvalues. Since

the beam is bedded, it has no rigid body motions and all eigenvalues of nonzero. Real eigenvalues occur as sets of two eigenvalues with opposite signs $\pm\lambda_\mathbb{R}$ and linearly independent eigenvectors $\boldsymbol{v}_{\mathbb{R}\pm} \in \mathbb{R}^6$. Complex eigenvalues appear as complex conjugates $\lambda_\mathbb{C}^\pm = \lambda_\mathfrak{R} \pm i\lambda_\mathfrak{I}$ with the corresponding complex eigenvectors $\boldsymbol{v}_\mathbb{C}^\pm = \boldsymbol{v}_\mathfrak{R} \pm i\boldsymbol{v}_\mathfrak{I}$ such that $\boldsymbol{v}_\mathbb{C}^\pm \in \mathbb{C}^6$ and $\boldsymbol{v}_\mathfrak{R}, \boldsymbol{v}_\mathfrak{I} \in \mathbb{R}^6$. Denoting the number of sets of real eigenvalue pairs as $N_\mathbb{R} \in \{0, \ldots, 3\}$ and the number of complex conjugate eigenvalue pairs as $N_\mathbb{C} \in \{0, \ldots, 3\}$ such that $N_\mathbb{R} + N_\mathbb{C} = 3$, the complementary solution is given by the linear combination

$$\begin{aligned}
\boldsymbol{z}_\mathrm{h}(x) = \sum_{n=1}^{N_\mathbb{R}} & C_{\mathbb{R}+}^{(n)} \exp\left(+\lambda_\mathbb{R}^{(n)} x\right) \boldsymbol{v}_{\mathbb{R}+}^{(n)} \\
& + C_{\mathbb{R}-}^{(n)} \exp\left(-\lambda_\mathbb{R}^{(n)} x\right) \boldsymbol{v}_{\mathbb{R}-}^{(n)} \\
+ \sum_{n=1}^{N_\mathbb{C}} & C_\mathfrak{R}^{(n)} \exp\left(\lambda_\mathfrak{R}^{(n)} x\right) \left[\boldsymbol{v}_\mathfrak{R}^{(n)} \cos\left(\lambda_\mathfrak{I}^{(n)} x\right) - \boldsymbol{v}_\mathfrak{I}^{(n)} \sin\left(\lambda_\mathfrak{I}^{(n)} x\right)\right] \\
& + C_\mathfrak{I}^{(n)} \exp\left(\lambda_\mathfrak{R}^{(n)} x\right) \left[\boldsymbol{v}_\mathfrak{R}^{(n)} \sin\left(\lambda_\mathfrak{I}^{(n)} x\right) + \boldsymbol{v}_\mathfrak{I}^{(n)} \cos\left(\lambda_\mathfrak{I}^{(n)} x\right)\right].
\end{aligned} \tag{S32}$$

The particular solution is obtained using the method of undetermined coefficients, which yields the constant vector

$$\boldsymbol{z}_\mathrm{p} = \begin{bmatrix} \frac{q_\mathrm{t}+p_\mathrm{t}}{k_\mathrm{t}} + \frac{h(h+t-2z_\mathrm{s})\,q_\mathrm{t}}{4\kappa A_{55}} & 0 & \frac{q_\mathrm{n}+p_\mathrm{n}}{k_\mathrm{n}} & 0 & \frac{(2z_\mathrm{s}-h-t)\,q_\mathrm{t}+(2h+t)\,p_\mathrm{t}}{2\kappa A_{55}} & 0 \end{bmatrix}^\top. \tag{S33}$$

Again, note the addition of surface loads p_n and p_t.[15] The general solution of the system

$$\boldsymbol{z}_\bullet(x) = \boldsymbol{z}_\mathrm{h}(x) + \boldsymbol{z}_\mathrm{p}, \tag{S34}$$

comprises six unknown coefficients $C_\bullet^{(n)}$ that must be identified from boundary and transmission conditions. It can be given in the matrix form

$$\boldsymbol{z}_\bullet(x) = \boldsymbol{Z}_\mathrm{h}(x)\,\boldsymbol{c}_\bullet + \boldsymbol{z}_\mathrm{p}, \tag{S35}$$

where $\boldsymbol{Z}_\mathrm{h} : \mathbb{R} \to \mathbb{R}^{6\times6}$ is a matrix-valued function with the summands of Eq. (S32) as column vectors and $\boldsymbol{c}_\bullet \in \mathbb{R}^6$ a vector containing the six free constants $C_\bullet^{(n)}$ according of Eq. (S32).

Layered segments without elastic foundation. To study situations where the weak layer has partially failed, the case of an unsupported slab must be considered. The situation can occur when the weak layer has collapsed or when a saw cut is introduced in a propagation saw test. Accounting for such cases allows for the use of the present model in failure models for anticrack nucleation[14] or growth.[22] If the slab is not supported by an elastic foundation, the general solution simplifies. In the equilibrium conditions (S15a) to (S15c), the normal and shear stress terms are omitted since no stresses act on the bottom side of the slab. The constitutive equations (S16a) and (S16b) remain the same.

Without elastic foundation, the equilibrium conditions (S15a) and (S15b) reduce to

$$0 = \frac{\mathrm{d}N(x)}{\mathrm{d}x} + q_\mathrm{t} + p_\mathrm{t}, \tag{S36a}$$

$$0 = \frac{\mathrm{d}V(x)}{\mathrm{d}x} + q_\mathrm{n} + p_\mathrm{n}, \tag{S36b}$$

$$0 = \frac{\mathrm{d}M(x)}{\mathrm{d}x} - V(x) + z_\mathrm{s} q_\mathrm{t} - \frac{h}{2} p_\mathrm{t}. \tag{S36c}$$

By adding and subtracting $\pm D_{11} w_0''(x)$ to the constitutive equation of the bending moment (S16a) and using the first derivative of the constitutive equation of the shear force (S16b)′, we obtain

$$M(x) = B_{11} u_0'(x) + \frac{D_{11}}{\kappa A_{55}} V'(x) - D_{11} w_0''(x). \tag{S37}$$

Differentiating twice and using the first derivatives of the equilibrium conditions, (S36b)′ and (S36c)′, yields

$$M''(x) = V'(x) = -(q_\mathrm{n} + p_\mathrm{n}) = B_{11} u_0'''(x) - D_{11} w_0''''(x). \tag{S38}$$

Adding and subtracting $\pm B_{11} w_0''$ to the constitutive equation of the normal force (S16a) and using the constitutive equation of the shear force (S16b), gives

$$N(x) = A_{11} u_0'(x) + \frac{B_{11}}{\kappa A_{55}} V'(x) - B_{11} w_0''(x). \tag{S39}$$

Differentiating this equation and, again, using the derivatives of the equilibrium conditions, (S36a)′ and (S36b)′, yields

$$N'(x) = -(q_\mathrm{t} + p_\mathrm{t}) = A_{11} u_0''(x) - B_{11} w_0'''(x). \tag{S40}$$

Solving the derivative of this equation for $u_0'''(x)$ and inserting it into Eq. (S38), yields an ordinary differential equation of fourth order for the vertical displacement

$$w_0''''(x) = -\frac{A_{11}}{B_{11}^2 - A_{11} D_{11}} (q_\mathrm{n} + p_\mathrm{n}). \tag{S41}$$

It can be solved readily by direct integration

$$\begin{aligned} w_0(x) &= c_1 + c_2 x + c_3 x^2 + c_4 x^3 \\ &\quad - \frac{A_{11}}{24(B_{11}^2 - A_{11} D_{11})} (q_\mathrm{n} + p_\mathrm{n}) x^4. \end{aligned} \tag{S42}$$

Solving Eq. (S40) for $u_0''(x)$, integrating twice and inserting the third derivative of the general solution for $w_0(x)$ (S42)′, yields the general solution for the tangential displacement of unsupported beams

$$\begin{aligned} u_0(x) &= c_5 + c_6 x + \frac{(6B_{11}c_4 - q_\mathrm{t} - p_\mathrm{t})}{2A_{11}} x^2 \\ &\quad - \frac{B_{11}}{6\left(B_{11}^2 - A_{11} D_{11}\right)} (q_\mathrm{n} + p_\mathrm{n}) x^3. \end{aligned} \tag{S43}$$

To obtain a solution of the cross-section rotation $\psi(x)$, we take the derivative of the constitutive equation for the bending moment (S16a)′ and insert it together with the constitutive equation of the shear force (S16b) into the equilibrium of moments (S36c). Solving this for $\psi(x)$ yields

$$\psi(x) = \frac{1}{\kappa A_{55}} \left(B_{11} u_0''(x) + D_{11} \psi''(x) + z_\mathrm{s} q_\mathrm{t} - \frac{h}{2} p_\mathrm{t}\right) - w_0'(x). \tag{S44}$$

Equation (S40) allows for eliminating $u_0''(x)$. In order to eliminate $\psi''(x)$, we insert the constitutive equation of the shear force (S16b) into the second derivative of the vertical equilibrium (S36b)″, which yields $\psi''(x) = -w_0'''(x)$ and we obtain

$$\begin{aligned} \psi(x) &= \frac{B_{11}^2 - A_{11} D_{11}}{\kappa A_{55} A_{11}} w_0'''(x) - w_0'(x) \\ &\quad + \left(z_\mathrm{s} - \frac{B_{11}}{A_{11}}\right) \frac{q_\mathrm{t}}{\kappa A_{55}} - \frac{h p_\mathrm{t}}{2\kappa A_{55}}, \end{aligned} \tag{S45}$$

which is fully defined through the solution for $w_0(x)$ (S42).

In order to assemble a global system of linear equations from boundary and transmission conditions between supported and unsupported beam segments, it is helpful to express the general solutions for both cases in the same form. For this purpose, we express vector of unknown functions (S25) used for the solution of supported beam segments through the general solutions (S42)

to (S45) for unsupported beam segments. This yields the matrix form

$$\boldsymbol{z}_\circ(x) = \boldsymbol{\mathcal{P}}(x)\,\boldsymbol{c}_\circ + \boldsymbol{p}(x), \tag{S46}$$

where

$$\boldsymbol{c}_\circ = \begin{bmatrix} C_\circ^{(1)} & C_\circ^{(2)} & \dots & C_\circ^{(6)} \end{bmatrix}^\intercal . \tag{S47}$$

is the vector of unknown coefficients,

$$\boldsymbol{\mathcal{P}}(x) = \begin{bmatrix} 0 & 0 & 0 & 3\frac{B_{11}}{A_{11}}x^2 & 1 & x \\ 0 & 0 & 0 & 6\frac{B_{11}}{A_{11}}x & 0 & 1 \\ 1 & x & x^2 & x^3 & 0 & 0 \\ 0 & 1 & 2x & 3x^2 & 0 & 0 \\ 0 & -1 & -2x & \frac{6K_0}{A_{11}\kappa A_{55}} - 3x^2 & 0 & 0 \\ 0 & 0 & -2 & -6x & 0 & 0 \end{bmatrix}, \tag{S48}$$

and

$$\boldsymbol{p}(x) = \begin{bmatrix} -\frac{q_\mathrm{t}+p_\mathrm{t}}{2A_{11}}x^2 - \frac{B_{11}}{6K_0}(q_\mathrm{n}+p_\mathrm{n})\,x^3 \\ -\frac{q_\mathrm{t}+p_\mathrm{t}}{A_{11}}x - \frac{B_{11}}{2K_0}(q_\mathrm{n}+p_\mathrm{n})\,x^2 \\ -\frac{A_{11}}{24K_0}(q_\mathrm{n}+p_\mathrm{n})\,x^4 \\ -\frac{A_{11}}{6K_0}(q_\mathrm{n}+p_\mathrm{n})\,x^3 \\ \frac{A_{11}}{6K_0}(q_\mathrm{n}+p_\mathrm{n})\,x^3 + \left(z_\mathrm{s} - \frac{B_{11}}{A_{11}}\right)\frac{q_\mathrm{t}}{\kappa A_{55}} - \frac{hp_\mathrm{t}}{2\kappa A_{55}} - \frac{q_\mathrm{n}+p_\mathrm{n}}{\kappa A_{55}}x \\ \frac{A_{11}}{2K_0}(q_\mathrm{n}+p_\mathrm{n})\,x^2 - \frac{q_\mathrm{n}+p_\mathrm{n}}{\kappa A_{55}} \end{bmatrix}, \tag{S49}$$

with $K_0 = B_{11}^2 - A_{11}D_{11}$.

Global system assembly. The general solutions presented above allow for the investigation of different systems composed of segments of supported and unsupported layered slabs. Possible configurations of interest are, e.g., skier-loaded snowpacks, skier-loaded snowpacks with a partially collapsed weak layer, or propagation saw test (PSTs) with an artificially introduced (sawed) edge crack. Assemblies of such configurations are illustrated in Fig. S9. Individual segments are connected through transmission conditions given in terms of displacements and section forces.

Stability tests are typically conducted on finite volumes with free ends that require vanishing section forces and moments

$$N = V = M = 0, \tag{S50}$$

as boundary conditions. Skier-induced loading is typically confined in a very small volume compared to the overall dimensions of the snowpack that extends over the entire slope. For the model, this corresponds to an unbounded domain where, all components of the solution converge to a constant at infinity. That is, at the boundaries, the complementary solution vector must vanish

$$\boldsymbol{z}_\mathrm{h} = 0, \tag{S51}$$

which yields constant displacements $\boldsymbol{z}(x) = \boldsymbol{z}_\mathrm{p}$, see Eq. (S33).

At interfaces between two segments (e.g., change from supported to unsupported), C^0-continuity of displacements and section forces is required and the transmission conditions read

$$\begin{aligned} \Delta u_0 &= 0, \quad \Delta w_0 = 0, \quad \Delta\psi = 0, \\ \Delta N &= 0, \quad \Delta V \;= 0, \quad \Delta M = 0, \end{aligned} \tag{S52}$$

where the Δ operator indicates the difference between left and right segments, i.e., $\Delta y = y_\mathrm{l} - y_\mathrm{r}$.

External concentrated forces (e.g., skiers) are introduced as discontinuities of the section forces. They are considered with their normal and tangential components F_n and F_t and with their resulting moment $M = -hF_\mathrm{t}/2$. They have to be accounted for in the form of the transmission conditions between two segments

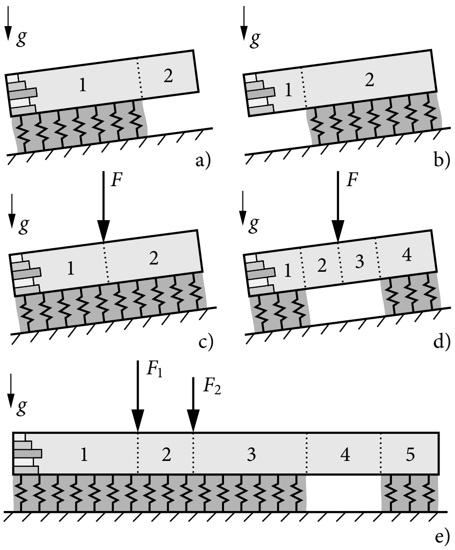

Supplementary Figure S9 | System assembly. Exemplary systems of interest assembled from supported and unsupported layered slabs with numbered segments: a) downslope PST, b) upslope PST, c) skier-loaded snowpack, d) partially fractured weak-layer, and e) layered slab loaded by multiple skiers with partially fractured weak-layer. Dotted lines indicate transmission conditions for the continuity of displacements and section forces.

$$\Delta N = F_\mathrm{t}, \quad \Delta V = F_\mathrm{n}, \quad \Delta M = -\frac{h}{2}F_\mathrm{t}, \tag{S53}$$

where again, the Δ operator expresses the difference between left and right segments. Therefore, at points of such loads the slab must always be split into segments to allow for the definition of the transmission conditions.

Inserting the general solutions (S35) and (S46) into the boundary and transmission conditions, yields equations that only depend on free constants. The set of equations can be assembled into a system of linear equations with $k = 6N_\mathrm{b}$ degrees of freedom, where N_b is the number of beam segments. In matrix form, the system reads

$$\boldsymbol{\Psi}\boldsymbol{c} = \boldsymbol{f}. \tag{S54}$$

Here, $\boldsymbol{\Psi} \in \mathbb{R}^{k\times k}$ is a square matrix of full rank, $\boldsymbol{c} \in \mathbb{R}^k$ is the vector of all free constants, and $\boldsymbol{f} \in \mathbb{R}^k$ is the right-hand-side vector that contains the particular solutions and displacement discontinuities induced by concentrated loads. With only k degrees of freedom, the system can be solved in real-time using standard methods such as Gaussian elimination or lower-upper (LU) decomposition.

Computation of displacements, stresses and energy release rates. Substituting the coefficients $C^{(n)}$ obtained from Eq. (S54) for each beam segment back into the general solutions (S35) and (S46), yields the vector $\boldsymbol{z}(x)$, which contains all slab displacement functions, see Eq. (S25).

Inserting the slab deformation solution into Eqs. (S14a) and (S14b), provides weak-layer normal and shear stresses, respectively. As discussed in the details of the mechanical model, weak-interface models do not allow for capturing highly localized stress concentrations (e.g., stress singularities) as they occur at crack tips. However, it is known that outside the direct vicinity of crack tips, the simplified weak-interface kinematics provide accurate displacement and stress solutions.[13]

The in-plane stresses σ_x, σ_z, and τ_{xz} within layers of the slab are obtained using the layers' constitutive equations and exploiting the equilibrium conditions.[16] Using Hooke's law and the identities $\varepsilon_x(x,z) = u'(x,z) = u_0'(x) + z\psi'(x)$, the axial layer normal stresses can be expressed in terms of slab displacements in the form

$$\sigma_x(x,z) = \frac{E(z)}{1-\nu(z)^2}\Big(u_0'(x) + z\psi'(x)\Big), \tag{S55}$$

where Young's modulus $E(z)$ and Poisson's ratio $\nu(z)$ are layer-wise, i.e., piecewise, constant in z-direction. Integrating the local equilibrium condition

$$0 = \frac{\partial\sigma_x}{\partial x} + \frac{\partial\tau_{xy}}{\partial y} + \frac{\partial\tau_{xz}}{\partial z}, \tag{S56}$$

with respect to z, where derivatives with respect to y vanish owing to the plane-strain assumption, yields the in-plane layer shear stress

$$\begin{aligned}\tau_{xz}(x,z) &= -\int \sigma_x'(x,z)\,\mathrm{d}z\\ &= -\int \frac{E(z)}{1-\nu(z)^2}\Big(u_0''(x) + z\psi''(x)\Big)\,\mathrm{d}z,\end{aligned} \tag{S57}$$

The second-order derivatives are obtained from the left-hand side of Eq. (S30) and integration with respect to z is performed using the trapezoidal rule. Again, integrating the equilibrium condition

$$0 = \frac{\partial\tau_{xz}}{\partial x} + \frac{\partial\tau_{yz}}{\partial y} + \frac{\partial\sigma_z}{\partial z}, \tag{S58}$$

with respect to z under the same assumptions, yields the interlayer normal stresses

$$\sigma_z(x,z) = -\int \tau_{xz}'(x,z)\,\mathrm{d}z. \tag{S59}$$

Here, differentiation is performed using difference quotients with consideration of discontinuities. Finally, maximum (σ_I) and minimum (σ_III) principal stresses are computed from

$$\sigma_{\mathrm{I,III}} = \frac{\sigma_x + \sigma_z}{2} \pm \sqrt{\left(\frac{\sigma_x - \sigma_z}{2}\right)^2 + \tau_{xz}^2}\,. \tag{S60}$$

The model can be used to determine the energy release rate of cracks. Here, we make use of the concept of anticracks,[23] that allows for studying failure of a weak layer in a snowpack exhibiting collapse.[24] As typical for fracture mechanics,[25] the symmetry of the displacement field around the crack tip can be used to identify symmetric (mode I) and antisymmetric deformations (mode II). We follow this convention to study mode I (crack closure) and mode II (crack sliding) energy release rates of anticracks. The energy release rate of cracks in weak interfaces can be given as

$$\mathcal{G}(a) = \mathcal{G}_\mathrm{I}(a) + \mathcal{G}_\mathrm{II}(a) = \frac{\sigma(a)^2}{2k_\mathrm{n}} + \frac{\tau(a)^2}{2k_\mathrm{t}}, \tag{S61}$$

where a denotes the crack-tip coordinate. The limitations of the weak-interface kinematics yield energy release rates that cannot capture very short cracks but, again, provide accurate results for cracks of a minimum length.[26] Cracks shorter than a few millimeters cannot be studied by the present approach.

Model validation

With reference to previous analysis of snowpack layering,[27,28] we use three-layered slabs proposed as schematic hardness profiles,[29] that are composed of soft, medium, and hard snow as benchmark slab configurations (Fig. S10). Assuming bonded slabs (e.g., rounded grains) and considering the density–hand hardness relations,[30] we assume densities of ρ = 350, 270, and 180 kg/m^3 for hard, medium, and soft snow layers with hand hardness indices pencil (P), four fingers (4F), and one finger (1F), respectively. From slab densities, we calculate the Young's modulus using a density-parametrization developed using acoustic wave propagation experiments[31] and improved using full-field displacement measurements[32]

$$E_\mathrm{sl}(\rho) = E_0\left(\frac{\rho}{\rho_0}\right)^\gamma, \tag{S62}$$

where $\gamma = 4.4$ accounts for density scaling and $E_0 = 6.5\cdot10^3$ MPa and $\rho_0 = 917\,\mathrm{kg/m^3}$ are Young's modulus and density of ice. Each slab layer is 12 cm thick and their individual material properties are given in Table S2. With reference to previous studies who report weak layer thickness between 0.2 and 3 cm,[33] we assume a weak-layer thickness of t = 2 cm. Following density measurements of surface hoar layers that report densities i) between 44 and 215 kg/m^3 with a mean of 102.5 kg/m^3 and ii) between 75 and 252 kg/m^3 with a mean of 132.4 kg/m^3 using two different measurement techniques, we assume a weak-layer density of ρ_wl = 100 kg/m^3, and a Young's modulus of E_wl = 0.15 MPa.[34] Other parameters are summarized in Table S3.

Finite element reference model. To validate the model, in particular with respect to different slab layerings, we compare the analytical solution to finite element analyses (FEA). The finite

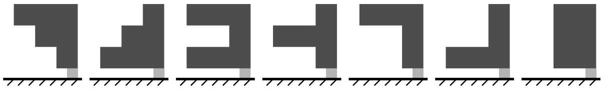

Supplementary Figure S10 | Benchmark profiles. Illustration of benchmark snow profiles used in the present work. Material properties of hard, medium, and soft slab layers (dark) and the weak layer (light) are given in Table S2. The weak layer is 2 cm thick and the slab layers have a thickness of 12 cm each.

Supplementary Table S2 | Snow profiles. Considered snow layers and their elastic properties with reference to three-layer slabs.[27]

Layer	Hand hardness index	Density ρ (kg/m^3)	Young's modulus E (MPa)	Poisson's ratio ν
Hard	P	350	93.8	0.25
Medium	1F	270	30.0	0.25
Soft	4F	180	5.0	0.25
Weak layer	F–	100	0.15	0.25

Supplementary Table S3 | Material properties. Material properties used throughout this work unless specified differently.

Property	Symbol	Value
Skier weight	m	80 kg
Slope angle	φ	38°
Slab thickness†	h	36 cm
Weak-layer thickness†	t	2 cm
Effective ouf-of-plane ski length	l_o	100 cm
Young's modulus weak layer	E_wl	0.15 MPa
Poisson's ratio	ν	0.25
Length of PST block	l_PST	250 cm
Length of PST cut	a_PST	50 cm

† Thicknesses given in slope-normal direction.

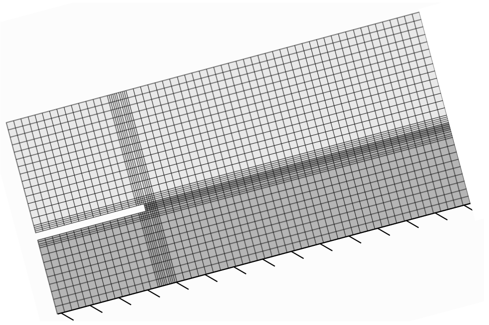

Supplementary Figure S11 | Finite element model used for validation. Discretization of a snowpack with slab and weak layer. Cracks are introduced by removing all weak layer elements. Skier loads are applied as vertical concentrated forces. Here, the case of a propagation saw test is shown as an example. The rigid base layer below the weak layer has a Young's modulus of $E_{\mathrm{bl}} = 10^{12}$ MPa.

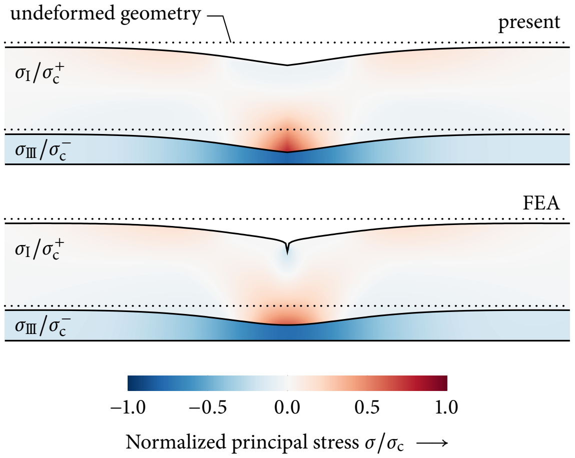

Supplementary Figure S12 | Stress field. Principal stresses and 200 times scaled snowpack deformations in the central 200 cm section of a skier-loaded snowpack comparing the present model (top) and the FEA reference model (bottom). In the homogeneous slab ▮H, maximum principal normal stresses σ_I (tension) normalized their tensile strength $\sigma_\mathrm{c}^+ = 9.1$ kPa are shown. In the weak layer we show minimum principal normal stresses σ_III (compression) normalized to an assumed weak layer compressive strength of $\sigma_\mathrm{c}^- = 2.6$ kPa. The weak-layer thickness is scaled by a factor of 4 for illustration.

element model is assembled from individual layers with unit out-of-plane width on an inclined slope (Fig. S11). Each layer is discretized using at least 10 eight-node biquadratic plane-strain continuum elements with reduced integration through its thickness. The lowest layer corresponds to the weak layer and rests on a rigid foundation. Weak-layer cracks are introduced by removing all weak-layer elements on the crack length a. We perform a static analysis of discrete geometric configurations with specific crack lengths a. Crack advance is not considered. The mesh is refined towards stress concentration such as crack tips and mesh convergence has been controlled carefully. The weight of the snowpack is introduced by providing the gravitational acceleration g and assigning each layer its corresponding density ρ. The load introduced by a skier is modeled as a concentrated force acting on the top of the slab. If skier loading is considered, the horizontal dimensions of the model are chosen large enough for all gradients to vanish. Typically 10 m suffice. Boundary conditions of PST experiments are free ends. In the FE model, the energy release rate of weak-layer cracks

$$\mathcal{G}_\mathrm{FE}(a) = -\frac{\partial \Pi(a)}{\partial a} \approx -\frac{\Pi(a+\Delta a) - \Pi(a-\Delta a)}{2\Delta a}, \tag{S63}$$

is computed using the central difference quotient to approximate the first derivative of the total potential Π with respect to a. The crack increment Δa corresponds to the element size and could be increased twofold or threefold without impacting computed values of $\mathcal{G}_\mathrm{FE}(a)$. Weak-layer stresses are evaluated in its vertical center.

Displacement and stress fields. Although visual representations of deformation and stress fields are limited to qualitative statements, they illustrate the principal responses of structures in different load cases. For this purpose, Fig. S12 compares principal stresses in a deformed slab-on-weak-layer system between present model and finite element reference solution. The system is loaded by the weight of the homogeneous slab ▮H and a concentrated force representing an 80 kg skier. Deformations are scaled by a factor of 200 and the weak-layer thickness by a factor of 4. In the slab, we show maximum principal normal stresses (tension) normalized to their tensile normal strength $\sigma_\mathrm{c}^+ = 9.1$ kPa obtained from the scaling law

$$\sigma_\mathrm{c}^+(\rho) = 240\ \mathrm{kPa}\left(\frac{\rho}{\rho_0}\right)^{2.44}, \tag{S64}$$

where $\rho_0 = 917\,\mathrm{kg/m^3}$ is the density of ice.[35] This illustrates the potential of tensile slab fracture. In the weak layer, minimum principal normal stresses (compression) normalized to their rapid-loading compressive strength $\sigma_\mathrm{c}^- = 2.6$ kPa[36] are shown, illustrating the potential for weak-layer collapse. We choose principal stresses for the visualization because they allow for the assessment of complex stress states by incorporating several stress components.

While the present model (Fig. S12, top panel) does not capture the highly localized stresses at the contact point between skier and slab observed in the FEA model (Fig. S12, bottom panel), the overall stress fields are in excellent agreement. This is consistent with Saint-Venant's principle, according to which the far-field effect of localized loads is independent of their asymptotic near-field behavior. The same holds for the displacement field. While the concentrated load introduces a dent in the slab's top surface, the overall deformations agree. With respect to the numerical reference, the present model renders displacement fields and both weak-layer and slab stresses well. Moreover, we can confirm the model assumption of constant stresses through the thickness of the weak layer.

Experimental validations are challenging since direct measurements of stresses are not possible and displacement measurements require considerable experimental effort. The latter can be recorded using digital image correlation (DIC).[32] From their analysis, we use the DIC-recorded displacement field of the first 1.3 m of a 3.0 ± 0.1 m long flat-field propagation saw test (Fig. S13, bottom panel). The PST was performed on January 7, 2019, had a slab thickness of $h = 46$ cm, a critical cut length of $a = 23 \pm 2$ cm, and the density profile shown in Fig. S13 (left panel) with a mean slab density of $\bar{\rho} = 111 \pm 6\,\mathrm{kg/m^3}$. From the density we computed individual layer stiffnesses according to Eq. (S62). Figure S13 compares both in-plane deformations of the snowpack (outlines) and the horizontal displacement fields (colorized overlay) obtained from the present model (top panel) and from DIC measurements (bottom panel). Deformations are scaled by a factor of 100, the weak-layer thickness by a factor of 10 for their visualization. In-plane slab and weak-layer deformations are in very good agreement. This is evident in both the deformed contours and in the colorized displacement field overlay. Since displacements are C^1-continuous across layer interfaces, the effect

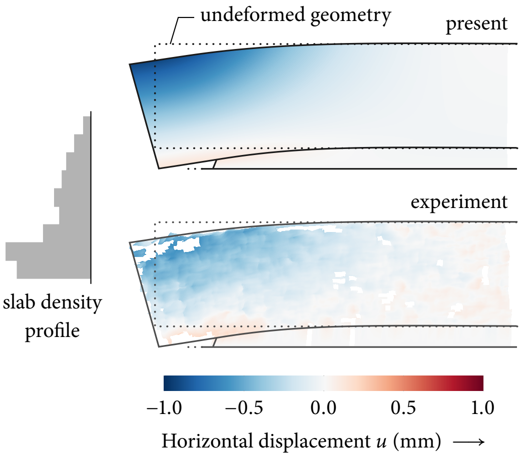

Supplementary Figure S13 | Displacement field. Horizontal displacement field of the first 1.3 m of a flat-field propagation saw test (PST) with an $a = 23$ cm cut into the $t = 1$ cm weak layer under a $h = 46$ cm slab. Comparison of the present model (top) with full-field digital image correlation measurements (bottom). White patches indicate missing data points. Deformations are scaled by a factor of 100 and the weak-layer thickness by a factor of 10 for illustration.

of layering is not directly visible in the displacement field. However, the slightly larger-than-expected tilt of the slab at its left end hints at a higher stiffness at the bottom of the slab and a compliant top section.

Weak-layer stresses and energy release rates. For all benchmark profiles illustrated in Fig. S10, weak-layer shear and normal stresses (τ, σ) obtained from the FEA model (dotted, light) and the present analytical solution (solid, dark) are compared in Fig. S14. We investigate a 38° inclined slope subjected to a concentrated force equivalent to the load of an 80 kg skier on an effective out-of-plane ski length of 1 m. The finite element reference model has a horizontal length of 10 m, of which the central 3 m are shown. The boundary conditions of the present model require the complementary solution (S32) to vanish, representing an infinite extension of the system.

Kinks in the model solution originate from the loading discontinuity introduced by the concentrated skier force. They are a direct result of the plate-theory modeling approach. The agreement with the FEA reference solution is close for all types of investigated profiles and layering effects on weak-layer stress distributions are well captured. Only for profile ⊐C, the present solution slightly underestimates the normal stress peak directly below the skier. This observation is not relevant for the prediction of weak-layer failure in a snow cover.[14] To study size effects present in any structure, a nonlocal evaluation of stresses must be used.[39–42] This has been discussed in detail by Leguillon[43], laying the foundation for the successful application of finite fracture mechanics approaches with weak-interface models.[44–46] Effects of bending stiffness (Fig. S14c vs. d) or bending–extension coupling (Fig. S14e vs. f) resulting from different layering orders, will be discussed in detail below.

A similar comparison of solutions for all profiles is given in Fig. S15, where total energy release rates (ERRs) of weak-layer anticracks in 38° inclined PST experiments are shown. Here, both models consider free boundaries of the 1.2 m long PST block. The structure is loaded by the weight of the slab and saw-introduced cracks are modeled by removing all weak-layer elements on the crack length a. This causes finite ERRs, even for very small cracks, because a finite amount of strain energy is removed from the

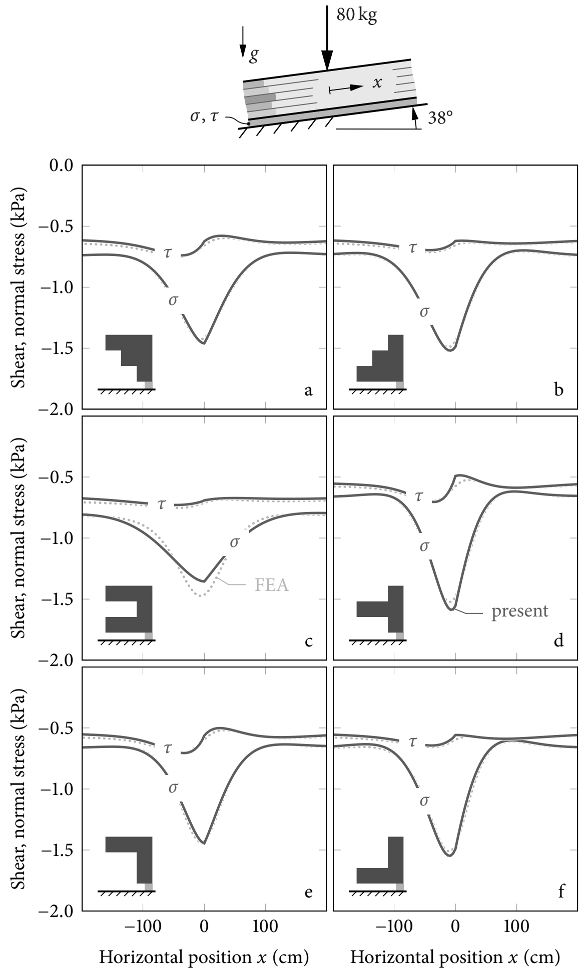

Supplementary Figure S14 | Weak-layer stresses. Weak-layer normal and shear stresses (σ, τ) owing to combined skier and snowpack-weight loading for the benchmark profiles illustrated in Fig. S10. The present solution (solid, dark) only slightly underestimates the maximum normal stresses with respect to the FEA reference (dotted, light) in the case of profile ⊐C. Material properties are given in Tables S2 and S3.

system with these elements. The ERR of a sharp crack is expected to vanish in the limit of zero crack length ($\ll 1$ cm).

The principal behavior of the ERR as a function of crack length is unaffected by the choice of profile. However, the different resulting stiffness and deformation properties influence the magnitude of the energy release rate considerably. For instance, between cases A and B, we observe a difference of almost 10 % (Fig. S15).

Figure S16 shows weak-layer fracture toughnesses determined from critical cut lengths of PSTs with layered slabs throughout the 2019 winter season using the present model.[32,37] The authors performed 21 tests on the same weak layer. While we observe small weak-layer fracture toughnesses at the beginning of January 2019, it quickly increases with the most significant precipitation event in mid January and then remains comparatively constant throughout the rest of the season.[32] For the purpose of validation of the present model, it is to note that all fracture toughnesses computed from the experiments lie within the bounds of the to date lowest[38] and highest[5] published values, 0.01 J/m^2 and 2.7 J/m^2, respectively.

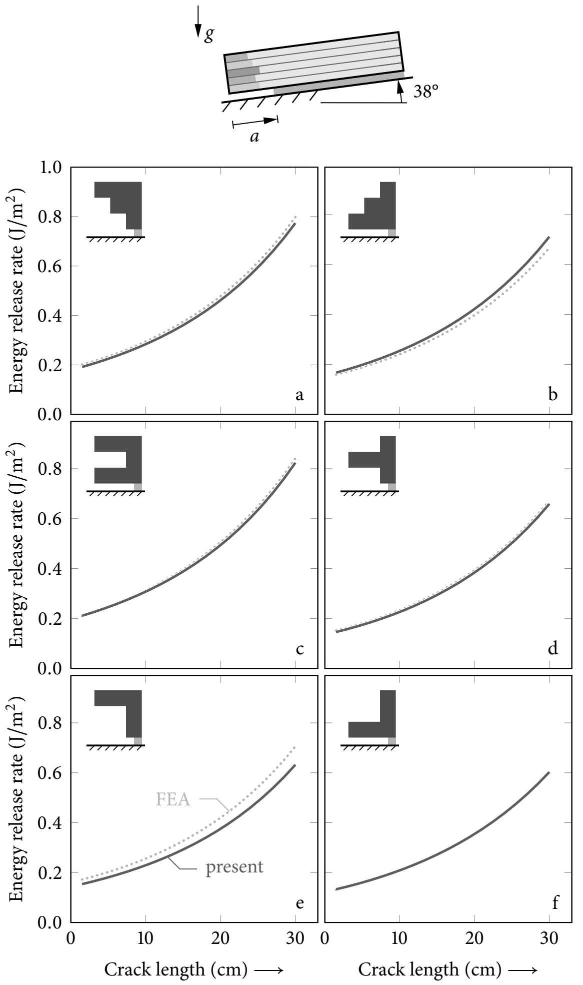

Supplementary Figure S15 | Anticrack energy release rate. Total energy release rates of weak-layer anticracks in 38° inclined PST experiments of 120 cm length with the benchmark profiles illustrated in Fig. S10. The present solution (solid, dark) and FEA reference (dotted, light) are in good agreement. Material properties are given in Tables S2 and S3.

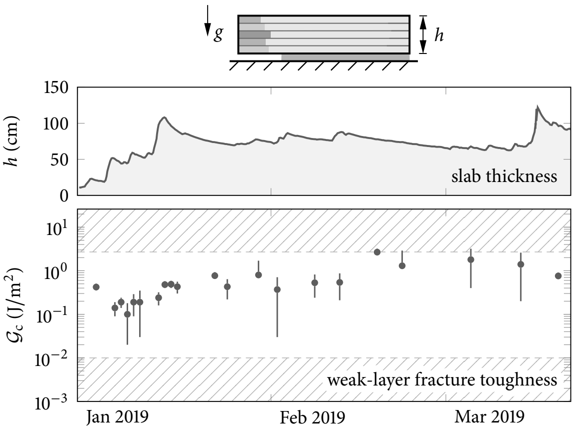

Supplementary Figure S16 | Weak-layer fracture toughness. Weak-layer fracture toughness determined with the present model from critical cut lengths of 21 flat-field propagation saw tests (PSTs) throughout the 2019 winter season on the same surface-hoar weak layer covered by a layered slab of changing thickness.[32,37] All results are within the hatched boundaries indicating the thus far lowest[38] and highest[5] published fracture toughness of weak layers, 0.01 J/m^2 and 2.7 J/m^2, respectively.

The present model can be classified as a structural-mechanics model as frequently employed in fracture mechanics. Structural models can be used to obtain effective quantities characterizing weak layers.[22] Effective quantities of fracture mechanics models are macroscopic quantities and always include microscopic mechanisms without further resolving their microscopic nature.[25] Identifying microstructural interactions in the fracture of highly-porous materials is a current active research topic.[47–49]

In comparison to previous studies that used similar concepts to model the structural response of layered snowpacks,[50–52] in our model we also consider slope normal deformations and the compliance of the weak layer. The latter effect has a substantial influence on the modeled energy release rate, which is central in this study.

Supplementary tables

Recorded data and literature data

Tables S4 and S5 list the experimental data with mean and standard deviation recorded at field sites A and B, respectively, between February 18 and March 10, 2022. Table S6 lists the literature data[5] evaluated for the present study.

Supplementary Table S4 | Field site A. Experimental data recorded at field site A between February 18 and March 3, 2022.

Date	Slope	Cut length	Fracture toughness	
	φ	a_c (cm)	$\mathcal{G}_{Ic}$ (J/m^2)	$\mathcal{G}_{IIc}$ (J/m^2)
Feb 18	0° ± 2°	10.90 ± 1.00	0.892 ± 0.173	0.022 ± 0.008
	10° ± 2°	15.50 ± 1.00	0.880 ± 0.143	0.061 ± 0.012
	15° ± 2°	14.75 ± 1.00	0.645 ± 0.112	0.070 ± 0.012
	20° ± 2°	16.75 ± 1.00	0.616 ± 0.106	0.101 ± 0.015
	25° ± 2°	19.00 ± 1.00	0.739 ± 0.126	0.157 ± 0.021
	35° ± 2°	20.00 ± 1.00	0.509 ± 0.100	0.218 ± 0.026
	40° ± 2°	23.50 ± 1.00	0.592 ± 0.117	0.305 ± 0.034
	45° ± 2°	26.50 ± 1.00	0.602 ± 0.126	0.391 ± 0.041
	50° ± 2°	28.75 ± 1.00	0.459 ± 0.111	0.447 ± 0.046
	50° ± 2°	31.00 ± 1.00	0.599 ± 0.136	0.510 ± 0.051
	55° ± 2°	34.00 ± 1.00	0.571 ± 0.147	0.614 ± 0.061
	60° ± 2°	38.50 ± 1.00	0.579 ± 0.175	0.772 ± 0.077
Feb 23	−49° ± 2°	9.20 ± 1.00	0.452 ± 0.059	0.098 ± 0.016
	14° ± 2°	14.00 ± 1.00	0.431 ± 0.070	0.042 ± 0.008
	25° ± 2°	14.25 ± 1.00	0.343 ± 0.064	0.087 ± 0.013
	36° ± 2°	27.75 ± 1.00	0.974 ± 0.149	0.304 ± 0.032
	47° ± 2°	28.00 ± 1.00	0.542 ± 0.106	0.367 ± 0.039
	53° ± 2°	35.75 ± 1.00	0.720 ± 0.151	0.567 ± 0.056
	57° ± 2°	38.00 ± 1.00	0.447 ± 0.117	0.612 ± 0.062
	61° ± 2°	37.50 ± 1.00	0.289 ± 0.094	0.606 ± 0.064

(continued on next page)

Table S4 | Field site A (continued)

Date	φ	a_c (cm)	$\mathcal{G}_{Ic}$ (J/m^2)	$\mathcal{G}_{IIc}$ (J/m^2)
	64° ± 2°	46.75 ± 1.00	0.346 ± 0.134	0.884 ± 0.091
Feb 24	−60° ± 2°	19.65 ± 1.00	0.867 ± 0.102	0.147 ± 0.026
	−50° ± 2°	16.55 ± 1.00	0.810 ± 0.094	0.109 ± 0.020
	−45° ± 2°	11.40 ± 1.00	0.551 ± 0.069	0.091 ± 0.016
	−40° ± 2°	16.75 ± 1.00	0.928 ± 0.108	0.064 ± 0.014
	−27° ± 2°	14.75 ± 1.00	0.802 ± 0.099	0.025 ± 0.008
	−18° ± 2°	14.55 ± 1.00	0.779 ± 0.101	0.006 ± 0.003
	−10° ± 2°	14.50 ± 1.00	0.705 ± 0.094	0.000 ± 0.000
	1° ± 2°	13.35 ± 1.00	0.510 ± 0.074	0.006 ± 0.003
	5° ± 2°	17.75 ± 1.00	0.720 ± 0.097	0.021 ± 0.006
	60° ± 2°	43.25 ± 1.00	0.391 ± 0.121	0.737 ± 0.076
Feb 25	24° ± 2°	19.50 ± 1.00	0.706 ± 0.106	0.126 ± 0.017
	28° ± 2°	22.90 ± 1.00	0.817 ± 0.119	0.182 ± 0.022
	32° ± 2°	24.70 ± 1.00	0.715 ± 0.108	0.222 ± 0.026
	35° ± 2°	25.75 ± 1.00	1.045 ± 0.157	0.285 ± 0.031
	37° ± 2°	23.00 ± 1.00	0.539 ± 0.091	0.240 ± 0.027
	42° ± 2°	28.50 ± 1.00	0.870 ± 0.143	0.371 ± 0.038
	47° ± 2°	28.45 ± 1.00	0.501 ± 0.098	0.386 ± 0.040
	53° ± 2°	33.75 ± 1.00	0.394 ± 0.094	0.515 ± 0.051
	53° ± 2°	31.25 ± 1.00	0.510 ± 0.112	0.482 ± 0.048
	56° ± 2°	34.00 ± 1.00	0.377 ± 0.096	0.542 ± 0.054
	57° ± 2°	49.00 ± 1.00	0.064 ± 0.060	1.015 ± 0.112
	65° ± 2°	42.50 ± 1.00	0.141 ± 0.072	0.778 ± 0.079
Mar 02	7° ± 2°	13.40 ± 1.00	0.458 ± 0.066	0.016 ± 0.005
	16° ± 2°	9.25 ± 1.00	0.248 ± 0.047	0.033 ± 0.007
	28° ± 2°	20.50 ± 1.00	0.496 ± 0.076	0.143 ± 0.019
	35° ± 2°	28.25 ± 1.00	0.875 ± 0.128	0.280 ± 0.030
	42° ± 2°	31.00 ± 1.00	0.699 ± 0.116	0.372 ± 0.038
	48° ± 2°	31.75 ± 1.00	0.437 ± 0.089	0.425 ± 0.044
	53° ± 2°	37.00 ± 1.00	0.555 ± 0.122	0.573 ± 0.056
	56° ± 2°	40.75 ± 1.00	0.386 ± 0.107	0.672 ± 0.067
	62° ± 2°	41.75 ± 1.00	0.270 ± 0.096	0.736 ± 0.074
	63° ± 2°	46.00 ± 1.00	0.333 ± 0.123	0.875 ± 0.087
	65° ± 2°	45.50 ± 1.00	0.175 ± 0.085	0.857 ± 0.087
Mar 03	8° ± 2°	15.75 ± 1.00	0.582 ± 0.080	0.024 ± 0.006
	11° ± 2°	16.25 ± 1.00	0.639 ± 0.090	0.038 ± 0.008
	17° ± 2°	20.00 ± 1.00	0.768 ± 0.104	0.079 ± 0.013
	26° ± 2°	22.25 ± 1.00	0.617 ± 0.089	0.142 ± 0.019
	32° ± 2°	26.00 ± 1.00	0.796 ± 0.115	0.227 ± 0.026
	36° ± 2°	24.75 ± 1.00	0.499 ± 0.081	0.236 ± 0.027
	42° ± 2°	31.25 ± 1.00	0.588 ± 0.102	0.367 ± 0.038
	46° ± 2°	36.50 ± 1.00	0.915 ± 0.156	0.513 ± 0.049
	50° ± 2°	38.50 ± 1.00	0.797 ± 0.153	0.589 ± 0.056
	55° ± 2°	40.25 ± 1.00	0.656 ± 0.147	0.672 ± 0.064
	59° ± 2°	44.50 ± 1.00	0.393 ± 0.122	0.800 ± 0.079

Supplementary Table S5 | Field site B. Experimental data recorded at field site B between March 7 and March 10, 2022.

Date	Slope	Cut length	Fracture toughness	

(continued on next page)

Table S5 | Field site B (continued)

Date	φ	a_c (cm)	$\mathcal{G}_{Ic}$ (J/m^2)	$\mathcal{G}_{IIc}$ (J/m^2)
	φ	a_c (cm)	$\mathcal{G}_{Ic}$ (J/m^2)	$\mathcal{G}_{IIc}$ (J/m^2)
Mar 07	59° ± 2°	29.75 ± 1.00	0.635 ± 0.247	0.755 ± 0.098
Mar 08	2° ± 2°	14.25 ± 1.00	0.575 ± 0.095	0.039 ± 0.009
	13° ± 2°	16.75 ± 1.00	0.696 ± 0.119	0.111 ± 0.019
	21° ± 2°	17.00 ± 1.00	0.542 ± 0.100	0.137 ± 0.021
	29° ± 2°	19.25 ± 1.00	0.403 ± 0.079	0.174 ± 0.023
	35° ± 2°	19.75 ± 1.00	0.326 ± 0.071	0.202 ± 0.026
	39° ± 2°	25.25 ± 1.00	0.584 ± 0.116	0.357 ± 0.041
	44° ± 2°	27.75 ± 1.00	0.556 ± 0.118	0.430 ± 0.048
	49° ± 2°	32.25 ± 1.00	0.711 ± 0.156	0.609 ± 0.066
	55° ± 2°	29.75 ± 1.00	0.196 ± 0.066	0.418 ± 0.050
	60° ± 2°	35.25 ± 1.00	0.404 ± 0.133	0.663 ± 0.080
Mar 09	−53° ± 2°	11.15 ± 1.00	0.603 ± 0.077	0.038 ± 0.010
	−48° ± 2°	9.10 ± 1.00	0.511 ± 0.069	0.035 ± 0.009
	−41° ± 2°	13.35 ± 1.00	0.823 ± 0.106	0.010 ± 0.005
	−22° ± 2°	13.75 ± 1.00	0.874 ± 0.122	0.002 ± 0.002
	−12° ± 2°	14.90 ± 1.00	0.877 ± 0.125	0.016 ± 0.006
	−1° ± 2°	9.90 ± 1.00	0.375 ± 0.067	0.011 ± 0.004
Mar 10	52° ± 2°	25.00 ± 1.00	0.347 ± 0.114	0.723 ± 0.086
	56° ± 2°	20.25 ± 1.00	0.127 ± 0.068	0.706 ± 0.091
	58° ± 2°	21.50 ± 1.00	0.082 ± 0.054	0.735 ± 0.095
	62° ± 2°	26.00 ± 1.00	0.029 ± 0.031	0.667 ± 0.086
	62° ± 2°	23.25 ± 1.00	0.120 ± 0.073	0.732 ± 0.095
	65° ± 2°	22.75 ± 1.00	0.006 ± 0.013	0.689 ± 0.095

Supplementary Table S6 | Historic data set. Literature data[5] on propagation saw tests evaluated for the present study, truncated at two digits.

#	Slope	Cut length	Fracture toughness	
	φ	a_c (cm)	$\mathcal{G}_{Ic}$ (J/m^2)	$\mathcal{G}_{IIc}$ (J/m^2)
1	0° ± 2°	19.98 ± 1.00	0.38 ± 0.05	0.00 ± 0.00
2	0° ± 2°	21.69 ± 1.00	0.13 ± 0.01	0.00 ± 0.00
3	0° ± 2°	52.04 ± 1.00	2.02 ± 0.22	0.01 ± 0.01
4	0° ± 2°	37.06 ± 1.00	0.99 ± 0.13	0.00 ± 0.00
5	0° ± 2°	34.41 ± 1.00	0.51 ± 0.06	0.00 ± 0.00
6	0° ± 2°	29.19 ± 1.00	0.51 ± 0.06	0.00 ± 0.00
7	0° ± 2°	31.81 ± 1.00	0.99 ± 0.12	0.00 ± 0.00
8	0° ± 2°	27.08 ± 1.00	0.18 ± 0.02	0.00 ± 0.00
9	0° ± 2°	33.93 ± 1.00	1.02 ± 0.12	0.00 ± 0.00
10	0° ± 2°	30.30 ± 1.00	0.22 ± 0.02	0.01 ± 0.00
11	0° ± 2°	30.53 ± 1.00	0.93 ± 0.12	0.00 ± 0.00
12	0° ± 2°	26.46 ± 1.00	0.18 ± 0.02	0.00 ± 0.00
13	0° ± 2°	33.18 ± 1.00	1.06 ± 0.13	0.00 ± 0.00
14	0° ± 2°	34.00 ± 1.00	0.29 ± 0.02	0.01 ± 0.00
15	0° ± 2°	16.96 ± 1.00	0.13 ± 0.01	0.00 ± 0.00
16	0° ± 2°	34.21 ± 1.00	0.30 ± 0.02	0.01 ± 0.00

(continued on next page)

Table S6 | Historic data set (continued)

#	φ	a_c (cm)	$\mathcal{G}_{Ic}$ (J/m^2)	$\mathcal{G}_{IIc}$ (J/m^2)
17	0° ± 2°	34.63 ± 1.00	0.30 ± 0.02	0.01 ± 0.00
18	0° ± 2°	31.53 ± 1.00	0.26 ± 0.02	0.00 ± 0.00
19	0° ± 2°	38.46 ± 1.00	0.37 ± 0.03	0.01 ± 0.00
20	0° ± 2°	29.54 ± 1.00	0.33 ± 0.04	0.00 ± 0.00
21	0° ± 2°	31.18 ± 1.00	0.42 ± 0.05	0.00 ± 0.00
22	0° ± 2°	53.80 ± 1.00	3.11 ± 0.38	0.00 ± 0.01
23	0° ± 2°	51.39 ± 1.00	2.93 ± 0.36	0.00 ± 0.01
24	0° ± 2°	28.60 ± 1.00	0.43 ± 0.04	0.00 ± 0.00
25	0° ± 2°	44.57 ± 1.00	0.99 ± 0.11	0.01 ± 0.00
26	0° ± 2°	32.10 ± 1.00	0.54 ± 0.07	0.00 ± 0.00
27	0° ± 2°	38.27 ± 1.00	1.16 ± 0.13	0.00 ± 0.00
28	0° ± 2°	16.54 ± 1.00	0.44 ± 0.06	0.00 ± 0.00
29	0° ± 2°	20.72 ± 1.00	0.19 ± 0.02	0.00 ± 0.00
30	0° ± 2°	23.16 ± 1.00	0.20 ± 0.02	0.00 ± 0.00
31	0° ± 2°	8.82 ± 1.00	0.05 ± 0.01	0.00 ± 0.00
32	0° ± 2°	15.91 ± 1.00	0.10 ± 0.01	0.00 ± 0.00
33	0° ± 2°	24.18 ± 1.00	0.26 ± 0.03	0.00 ± 0.00
34	0° ± 2°	24.63 ± 1.00	0.27 ± 0.03	0.00 ± 0.00
35	0° ± 2°	23.35 ± 1.00	0.25 ± 0.03	0.00 ± 0.00
36	0° ± 2°	29.32 ± 1.00	0.35 ± 0.04	0.01 ± 0.00
37	0° ± 2°	26.91 ± 1.00	0.30 ± 0.03	0.00 ± 0.00
38	0° ± 2°	32.91 ± 1.00	0.39 ± 0.04	0.01 ± 0.00
39	0° ± 2°	31.90 ± 1.00	0.37 ± 0.03	0.01 ± 0.00
40	0° ± 2°	33.27 ± 1.00	0.39 ± 0.04	0.01 ± 0.00
41	0° ± 2°	27.45 ± 1.00	0.31 ± 0.03	0.00 ± 0.00
42	0° ± 2°	31.86 ± 1.00	0.38 ± 0.04	0.00 ± 0.00
43	0° ± 2°	31.43 ± 1.00	0.68 ± 0.08	0.00 ± 0.00
44	0° ± 2°	29.79 ± 1.00	0.32 ± 0.03	0.01 ± 0.00
45	0° ± 2°	34.47 ± 1.00	0.45 ± 0.04	0.01 ± 0.00
46	0° ± 2°	35.42 ± 1.00	0.49 ± 0.05	0.01 ± 0.00
47	0° ± 2°	21.80 ± 1.00	0.19 ± 0.02	0.00 ± 0.00
48	0° ± 2°	23.19 ± 1.00	0.22 ± 0.03	0.00 ± 0.00
49	0° ± 2°	36.76 ± 1.00	0.80 ± 0.10	0.00 ± 0.00
50	0° ± 2°	23.11 ± 1.00	0.32 ± 0.04	0.00 ± 0.00
51	0° ± 2°	23.62 ± 1.00	0.26 ± 0.03	0.00 ± 0.00
52	0° ± 2°	18.71 ± 1.00	0.36 ± 0.04	0.00 ± 0.00
53	0° ± 2°	24.43 ± 1.00	0.48 ± 0.06	0.00 ± 0.00
54	0° ± 2°	28.36 ± 1.00	0.56 ± 0.09	0.00 ± 0.00
55	0° ± 2°	47.10 ± 1.00	2.36 ± 0.29	0.00 ± 0.00
56	0° ± 2°	23.00 ± 1.00	0.45 ± 0.06	0.00 ± 0.00
57	0° ± 2°	38.22 ± 1.00	0.68 ± 0.07	0.00 ± 0.00
58	0° ± 2°	25.30 ± 1.00	0.79 ± 0.09	0.00 ± 0.00
59	0° ± 2°	34.75 ± 1.00	1.10 ± 0.12	0.00 ± 0.00
60	0° ± 2°	35.44 ± 1.00	1.38 ± 0.16	0.00 ± 0.00
61	0° ± 2°	38.39 ± 1.00	1.50 ± 0.17	0.00 ± 0.00
62	0° ± 2°	20.98 ± 1.00	0.86 ± 0.11	0.00 ± 0.00
63	0° ± 2°	29.36 ± 1.00	0.61 ± 0.07	0.00 ± 0.00
64	0° ± 2°	46.27 ± 1.00	1.17 ± 0.11	0.01 ± 0.01
65	0° ± 2°	33.99 ± 1.00	0.67 ± 0.07	0.01 ± 0.00
66	0° ± 2°	36.80 ± 1.00	0.80 ± 0.09	0.00 ± 0.00
67	0° ± 2°	29.06 ± 1.00	0.55 ± 0.06	0.00 ± 0.00
68	0° ± 2°	27.58 ± 1.00	0.52 ± 0.06	0.00 ± 0.00
69	0° ± 2°	30.56 ± 1.00	0.59 ± 0.07	0.00 ± 0.00

(continued on next page)

Table S6 | Historic data set (continued)

#	φ	a_c (cm)	$\mathcal{G}_{Ic}$ (J/m^2)	$\mathcal{G}_{IIc}$ (J/m^2)
70	0° ± 2°	38.69 ± 1.00	0.72 ± 0.07	0.00 ± 0.00
71	0° ± 2°	41.01 ± 1.00	1.43 ± 0.16	0.00 ± 0.00
72	0° ± 2°	39.06 ± 1.00	0.71 ± 0.07	0.00 ± 0.00
73	0° ± 2°	37.61 ± 1.00	1.07 ± 0.12	0.01 ± 0.00
74	0° ± 2°	46.29 ± 1.00	1.68 ± 0.20	0.01 ± 0.01
75	0° ± 2°	47.90 ± 1.00	1.39 ± 0.14	0.01 ± 0.01
76	0° ± 2°	56.34 ± 1.00	2.18 ± 0.23	0.01 ± 0.01
77	0° ± 2°	33.61 ± 1.00	1.28 ± 0.16	0.00 ± 0.00
78	0° ± 2°	48.23 ± 1.00	1.87 ± 0.21	0.00 ± 0.00
79	0° ± 2°	52.43 ± 1.00	3.55 ± 0.46	0.00 ± 0.01
80	0° ± 2°	69.26 ± 1.00	6.22 ± 0.75	0.00 ± 0.01
81	0° ± 2°	58.13 ± 1.00	4.12 ± 0.50	0.01 ± 0.01
82	0° ± 2°	71.48 ± 1.00	6.25 ± 0.74	0.01 ± 0.01
83	0° ± 2°	77.64 ± 1.00	15.16 ± 2.00	0.00 ± 0.01
84	0° ± 2°	40.21 ± 1.00	1.40 ± 0.16	0.00 ± 0.00
85	0° ± 2°	32.21 ± 1.00	0.29 ± 0.03	0.00 ± 0.00
86	0° ± 2°	37.94 ± 1.00	0.78 ± 0.07	0.01 ± 0.00
87	0° ± 2°	24.82 ± 1.00	0.46 ± 0.05	0.00 ± 0.00
88	0° ± 2°	32.81 ± 1.00	0.65 ± 0.06	0.00 ± 0.00
89	0° ± 2°	27.86 ± 1.00	0.41 ± 0.05	0.00 ± 0.00
90	0° ± 2°	36.42 ± 1.00	0.63 ± 0.07	0.00 ± 0.00
91	0° ± 2°	26.62 ± 1.00	0.36 ± 0.04	0.00 ± 0.00
92	0° ± 2°	23.73 ± 1.00	0.29 ± 0.03	0.00 ± 0.00
93	0° ± 2°	16.65 ± 1.00	0.20 ± 0.02	0.00 ± 0.00
94	0° ± 2°	31.46 ± 1.00	0.43 ± 0.04	0.00 ± 0.00
95	0° ± 2°	36.73 ± 1.00	0.54 ± 0.05	0.01 ± 0.00
96	0° ± 2°	35.24 ± 1.00	0.50 ± 0.05	0.00 ± 0.00
97	0° ± 2°	28.25 ± 1.00	0.53 ± 0.05	0.00 ± 0.00
98	0° ± 2°	32.99 ± 1.00	0.54 ± 0.06	0.00 ± 0.00
99	0° ± 2°	30.27 ± 1.00	0.60 ± 0.06	0.00 ± 0.00
100	0° ± 2°	28.38 ± 1.00	0.59 ± 0.06	0.00 ± 0.00
101	0° ± 2°	27.54 ± 1.00	0.54 ± 0.06	0.00 ± 0.00
102	0° ± 2°	32.86 ± 1.00	0.66 ± 0.07	0.00 ± 0.00
103	0° ± 2°	60.72 ± 1.00	1.22 ± 0.07	0.09 ± 0.01
104	0° ± 2°	20.07 ± 1.00	0.41 ± 0.05	0.00 ± 0.00
105	0° ± 2°	32.31 ± 1.00	0.64 ± 0.06	0.00 ± 0.00
106	0° ± 2°	41.95 ± 1.00	2.09 ± 0.29	0.00 ± 0.00
107	0° ± 2°	44.08 ± 1.00	0.56 ± 0.04	0.03 ± 0.00
108	−8° ± 2°	16.14 ± 1.00	0.17 ± 0.02	0.00 ± 0.00
109	−8° ± 2°	25.55 ± 1.00	0.27 ± 0.02	0.00 ± 0.00
110	−9° ± 2°	25.63 ± 1.00	0.27 ± 0.02	0.00 ± 0.00
111	−10° ± 2°	32.32 ± 1.00	0.63 ± 0.07	0.00 ± 0.00
112	−10° ± 2°	30.30 ± 1.00	0.39 ± 0.03	0.00 ± 0.00
113	−10° ± 2°	32.82 ± 1.00	0.73 ± 0.07	0.00 ± 0.00
114	−10° ± 2°	29.41 ± 1.00	0.59 ± 0.06	0.00 ± 0.00
115	−10° ± 2°	31.14 ± 1.00	0.69 ± 0.07	0.00 ± 0.00
116	−10° ± 2°	37.14 ± 1.00	0.98 ± 0.10	0.00 ± 0.00
117	−10° ± 2°	31.80 ± 1.00	0.48 ± 0.04	0.00 ± 0.00
118	−10° ± 2°	25.67 ± 1.00	0.49 ± 0.06	0.00 ± 0.00
119	−10° ± 2°	24.88 ± 1.00	0.53 ± 0.06	0.00 ± 0.00
120	−10° ± 2°	28.89 ± 1.00	0.60 ± 0.07	0.00 ± 0.00
121	−10° ± 2°	49.62 ± 1.00	0.89 ± 0.06	0.00 ± 0.00
122	−10° ± 2°	30.65 ± 1.00	0.52 ± 0.05	0.00 ± 0.00

(continued on next page)

Table S6 | Historic data set (continued)

#	φ	a_c (cm)	$\mathcal{G}_{Ic}$ (J/m^2)	$\mathcal{G}_{IIc}$ (J/m^2)
123	−13° ± 2°	17.31 ± 1.00	0.19 ± 0.02	0.00 ± 0.00
124	−15° ± 2°	17.13 ± 1.00	0.18 ± 0.02	0.00 ± 0.00
125	−15° ± 2°	17.15 ± 1.00	0.17 ± 0.02	0.00 ± 0.00
126	−17° ± 2°	19.43 ± 1.00	0.20 ± 0.02	0.00 ± 0.00
127	−18° ± 2°	29.08 ± 1.00	0.80 ± 0.08	0.01 ± 0.00
128	−18° ± 2°	53.46 ± 1.00	4.55 ± 0.57	0.01 ± 0.01
129	−18° ± 2°	42.11 ± 1.00	2.90 ± 0.38	0.01 ± 0.01
130	−18° ± 2°	37.17 ± 1.00	1.03 ± 0.10	0.00 ± 0.00
131	−18° ± 2°	21.40 ± 1.00	0.29 ± 0.03	0.00 ± 0.00
132	−18° ± 2°	56.46 ± 1.00	3.97 ± 0.39	0.08 ± 0.02
133	−19° ± 2°	19.72 ± 1.00	0.18 ± 0.02	0.01 ± 0.00
134	−19° ± 2°	36.74 ± 1.00	0.32 ± 0.02	0.00 ± 0.00
135	−19° ± 2°	64.28 ± 1.00	1.83 ± 0.11	0.00 ± 0.00
136	−20° ± 2°	56.61 ± 1.00	2.26 ± 0.22	0.02 ± 0.01
137	−20° ± 2°	37.04 ± 1.00	1.08 ± 0.11	0.02 ± 0.01
138	−20° ± 2°	22.70 ± 1.00	0.26 ± 0.02	0.00 ± 0.00
139	−20° ± 2°	33.21 ± 1.00	0.47 ± 0.03	0.00 ± 0.00
140	−20° ± 2°	18.30 ± 1.00	0.20 ± 0.02	0.01 ± 0.00
141	−20° ± 2°	25.11 ± 1.00	0.33 ± 0.04	0.01 ± 0.00
142	−20° ± 2°	45.98 ± 1.00	1.28 ± 0.12	0.02 ± 0.01
143	−20° ± 2°	50.44 ± 1.00	2.11 ± 0.22	0.02 ± 0.01
144	−20° ± 2°	56.14 ± 1.00	2.49 ± 0.26	0.02 ± 0.01
145	−20° ± 2°	60.99 ± 1.00	4.58 ± 0.52	0.03 ± 0.01
146	−21° ± 2°	23.12 ± 1.00	0.17 ± 0.02	0.00 ± 0.00
147	−21° ± 2°	17.00 ± 1.00	0.11 ± 0.01	0.00 ± 0.00
148	−21° ± 2°	32.07 ± 1.00	1.61 ± 0.17	0.05 ± 0.01
149	−21° ± 2°	22.58 ± 1.00	0.15 ± 0.01	0.00 ± 0.00
150	−21° ± 2°	18.01 ± 1.00	0.12 ± 0.01	0.00 ± 0.00
151	−21° ± 2°	35.25 ± 1.00	0.38 ± 0.03	0.00 ± 0.00
152	−21° ± 2°	19.23 ± 1.00	0.23 ± 0.02	0.01 ± 0.00
153	−21° ± 2°	6.46 ± 1.00	0.04 ± 0.01	0.00 ± 0.00
154	−21° ± 2°	6.36 ± 1.00	0.04 ± 0.01	0.00 ± 0.00
155	−21° ± 2°	26.25 ± 1.00	1.12 ± 0.11	0.05 ± 0.01
156	−21° ± 2°	41.73 ± 1.00	1.86 ± 0.19	0.05 ± 0.01
157	−22° ± 2°	19.97 ± 1.00	0.13 ± 0.01	0.00 ± 0.00
158	−22° ± 2°	16.85 ± 1.00	0.19 ± 0.02	0.00 ± 0.00
159	−23° ± 2°	42.76 ± 1.00	0.37 ± 0.03	0.00 ± 0.00
160	−24° ± 2°	31.09 ± 1.00	0.39 ± 0.04	0.02 ± 0.00
161	−25° ± 2°	15.46 ± 1.00	0.07 ± 0.01	0.00 ± 0.00
162	−25° ± 2°	26.50 ± 1.00	0.33 ± 0.03	0.00 ± 0.00
163	−26° ± 2°	26.53 ± 1.00	0.21 ± 0.02	0.00 ± 0.00
164	−26° ± 2°	30.78 ± 1.00	0.26 ± 0.02	0.00 ± 0.00
165	−27° ± 2°	23.61 ± 1.00	0.52 ± 0.04	0.02 ± 0.00
166	−27° ± 2°	25.41 ± 1.00	0.55 ± 0.04	0.02 ± 0.00
167	−28° ± 2°	27.67 ± 1.00	0.12 ± 0.01	0.00 ± 0.00
168	−28° ± 2°	29.35 ± 1.00	0.14 ± 0.01	0.00 ± 0.00
169	−28° ± 2°	29.84 ± 1.00	0.17 ± 0.01	0.00 ± 0.00
170	−28° ± 2°	32.07 ± 1.00	0.54 ± 0.04	0.00 ± 0.00
171	−28° ± 2°	41.74 ± 1.00	0.57 ± 0.07	0.00 ± 0.00
172	−30° ± 2°	44.29 ± 1.00	0.81 ± 0.06	0.02 ± 0.01
173	−31° ± 2°	36.61 ± 1.00	0.33 ± 0.03	0.01 ± 0.00
174	−32° ± 2°	40.96 ± 1.00	0.92 ± 0.06	0.01 ± 0.00
175	−33° ± 2°	66.99 ± 1.00	1.66 ± 0.13	0.02 ± 0.01

(continued on next page)

Table S6 | Historic data set (continued)

#	φ	a_c (cm)	$\mathcal{G}_{Ic}$ (J/m^2)	$\mathcal{G}_{IIc}$ (J/m^2)
176	−34° ± 2°	66.21 ± 1.00	3.28 ± 0.35	0.05 ± 0.01
177	−34° ± 2°	60.86 ± 1.00	1.07 ± 0.07	0.00 ± 0.00
178	−35° ± 2°	39.18 ± 1.00	0.31 ± 0.02	0.00 ± 0.00
179	−35° ± 2°	23.03 ± 1.00	0.43 ± 0.03	0.02 ± 0.00
180	−35° ± 2°	20.70 ± 1.00	0.37 ± 0.03	0.02 ± 0.00
181	−36° ± 2°	44.30 ± 1.00	0.99 ± 0.07	0.07 ± 0.02
182	−36° ± 2°	37.65 ± 1.00	1.33 ± 0.13	0.12 ± 0.02

Supplementary references

[1] Fierz, C. *et al.* The international classification for seasonal snow on the ground. *IHP-VII Technical Documents in Hydrology* **83** (2009).

[2] van Herwijnen, A. & Jamieson, B. High-speed photography of fractures in weak snowpack layers. *Cold Regions Science and Technology* **43**, 71–82 (2005).

[3] Gauthier, D. & Jamieson, B. Towards a field test for fracture propagation propensity in weak snowpack layers. *Journal of Glaciology* **52**, 164–168 (2006).

[4] Sigrist, C., Schweizer, J., Schindler, H.-J. & Dual, J. The energy release rate of mode II fractures in layered snow samples. *International Journal of Fracture* **139**, 461–475 (2006). URL http://link.springer.com/10.1007/s10704-006-6580-9.

[5] van Herwijnen, A. *et al.* Estimating the effective elastic modulus and specific fracture energy of snowpack layers from field experiments. *Journal of Glaciology* **62**, 997–1007 (2016).

[6] Boggs, P. T., Byrd, R. H. & Schnabel, R. A Stable and Efficient Algorithm for Nonlinear Orthogonal Distance Regression. *SIAM Journal on Scientific and Statistical Computing* **8**, 1052–1078 (1987).

[7] Boggs, P. T., Donaldson, J. R., Byrd, R. h. & Schnabel, R. B. Algorithm 676: ODRPACK: software for weighted orthogonal distance regression. *ACM Transactions on Mathematical Software* **15**, 348–364 (1989).

[8] Boggs, P. T., Byrd, R. H., Rogers, J. E. & Schnabel, R. B. User's Reference Guide for ODRPACK Version 2.01 Software for Weighted Orthogonal Distance Regression. *U.S. Department of Commerce* 99 (1992).

[9] Mantič, V., Távara, L., Blázquez, A., Graciani, E. & París, F. A linear elastic-brittle interface model: application for the onset and propagation of a fibre-matrix interface crack under biaxial transverse loads. *International Journal of Fracture* **195**, 15–38 (2015). URL http://link.springer.com/10.1007/s10704-015-0043-0.

[10] Benzeggagh, M. L. & Kenane, M. Measurement of mixed-mode delamination fracture toughness of unidirectional glass/epoxy composites with mixed-mode bending apparatus. *Composites Science and Technology* **56**, 439–449 (1996).

[11] Hutchinson, J. W. & Suo, Z. Mixed Mode Cracking in Layered Materials. In *Journal of Applied Mechanics*, vol. 27, 63–191 (1991).

[12] Marşavina, L. & Linul, E. Fracture toughness of rigid polymeric foams: A review. *Fatigue & Fracture of Engineering Materials & Structures* **43**, 2483–2514 (2020).

[13] Rosendahl, P. L. & Weißgraeber, P. Modeling snow slab avalanches caused by weak-layer failure – Part 1: Slabs on compliant and collapsible weak layers. *The Cryosphere* **14**, 115–130 (2020). URL https://www.the-cryosphere.net/14/115/2020/.

[14] Rosendahl, P. L. & Weißgraeber, P. Modeling snow slab avalanches caused by weak-layer failure – Part 2: Coupled mixed-mode criterion for skier-triggered anticracks. *The Cryosphere* **14**, 131–145 (2020). URL https://www.the-cryosphere.net/14/131/2020/.

[15] Weißgraeber, P. & Rosendahl, P. L. A closed-form model for layered snow slabs. *The Cryosphere Discussions* **2022**, 1–37 (2022).

[16] Reddy, J. N. *Mechanics of Laminated Composite Plates and Shells: Theory and Analysis* (CRC Press, Boca Raton, 2003), 2nd edn.

[17] Goland, M. & Reissner, E. The stresses in cemented joints. *Journal of Applied Mechanics* **11**, A17–A27 (1944).

[18] Lenci, S. Analysis of a crack at a weak interface. *International Journal of Fracture* **108**, 275–290 (2001).

[19] Krenk, S. Energy release rate of symmetric adhesive joints. *Engineering Fracture Mechanics* **43**, 549–559 (1992).

[20] Stein, N., Weißgraeber, P. & Becker, W. A model for brittle failure in adhesive lap joints of arbitrary joint configuration. *Composite Structures* **133**, 707–718 (2015).

[21] Klarmann, R. & Schweizerhof, K. A Priori Verbesserung von Schubkorrekturfaktoren zur Berechnung von geschichteten anisotropen Schalentragwerken. *Archive of Applied Mechanics* **63**, 73–85 (1993).

[22] Bergfeld, B. *et al.* Dynamic crack propagation in weak snowpack layers: Insights from high-resolution, high-speed photography. *The Cryosphere Discussions* **2021**, 1–21 (2021).

[23] Fletcher, R. C. & Pollard, D. D. Anticrack model for pressure solution surfaces. *Geology* **9**, 419–424 (1981).

[24] Heierli, J., Gumbsch, P. & Zaiser, M. Anticrack nucleation as triggering mechanism for snow slab avalanches. *Science* **321**, 240–243 (2008).

[25] Broberg, K. B. The near-tip field at high crack velocities. *International Journal of Fracture* **39**, 1–13 (1989).

[26] Hübsch, J. D., Rosendahl, P. L. & Mittelstedt, C. An analytical failure model for pressurized blister tests of thermally loaded composite laminates. *Composites Part B: Engineering* **214**, 108588 (2021).

[27] Habermann, M., Schweizer, J. & Jamieson, B. Influence of snowpack layering on human-triggered snow slab avalanche release. *Cold Regions Science and Technology* **54**, 176–182 (2008).

[28] Monti, F., Gaume, J., van Herwijnen, A. & Schweizer, J. Snow instability evaluation: calculating the skier-induced stress in a multi-layered snowpack. *Natural Hazards and Earth System Sciences Discussions* **3**, 4833–4869 (2015).

[29] Schweizer, J. & Wiesinger, T. Snow profile interpretation for stability evaluation. *Cold Regions Science and Technology* **33**, 179–188 (2001).

[30] Geldsetzer, T. & Jamieson, B. Estimating dry snow density from grain form and hand hardness. In *International Snow Science Workshop*, 121–127 (2000).

[31] Gerling, B., Löwe, H. & van Herwijnen, A. Measuring the Elastic Modulus of Snow. *Geophysical Research Letters* **44**, 11,088–11,096 (2017).

[32] Bergfeld, B. *et al.* Temporal evolution of crack propagation characteristics in a weak snowpack layer: conditions of crack arrest and sustained propagation. *Natural Hazards and Earth System Sciences* **23**, 293–315 (2023).

[33] Jamieson, B. & Schweizer, J. Texture and strength changes of buried surface-hoar layers with implications for dry snow-slab avalanche release. *Journal of Glaciology* **46**, 151–160 (2000).

[34] Föhn, P. M. B. Simulation of surface-hoar layers for snow-cover models. *Annals of Glaciology* **32**, 19–26 (2001).

[35] Sigrist, C. *Measurement of fracture mechanical properties of snow and application to dry snow slab avalanche release*. Ph.D. thesis, ETH Zürich (2006).

[36] Reiweger, I., Gaume, J. & Schweizer, J. A new mixed-mode failure criterion for weak snowpack layers. *Geophysical Research Letters* **42**, 1427–1432 (2015). URL http://doi.wiley.com/10.1002/2014GL062780.

[37] Bergfeld, B., van Herwijnen, A. & Schweizer, J. Time series data on dynamic crack propagation in long propagation saw tests. *EnviDat* (2023).

[38] Gauthier, D. & Jamieson, B. On the Sustainability and Arrest of Weak Layer Fracture in Whumpfs and Avalanches. In *Proceedings of the International Snow Science Workshop*, vol. 1, 224–231 (2010).

[39] Neuber, H. Theorie der technischen Formzahl. *Forschung auf dem Gebiete des Ingenieurwesens* **7**, 271–274 (1936).

[40] Peterson, R. E. Methods of correlating data from fatigue tests of stress concentration specimens. In *Stephen Timoshenko Anniversary Volume*, 179–183 (Macmillan, New York, 1938).

[41] Waddoups, M. E., Eisenmann, J. R. & Kaminski, B. E. Macroscopic Fracture Mechanics of Advanced Composite Materials. *Journal of Composite Materials* **5**, 446–454 (1971).

[42] Sih, G. C. Strain-energy-density factor applied to mixed mode crack problems. *International Journal of Fracture* **10**, 305–321 (1974). I248-1974-003.

[43] Leguillon, D. Strength or toughness? A criterion for crack onset at a notch. *European Journal of Mechanics – A/Solids* **21**, 61–72 (2002).

[44] Cornetti, P., Mantič, V. & Carpinteri, A. Finite Fracture Mechanics at elastic interfaces. *International Journal of Solids and Structures* **49**, 1022–1032 (2012).

[45] Weißgraeber, P. & Becker, W. Finite Fracture Mechanics model for mixed mode fracture in adhesive joints. *International Journal of Solids and Structures* **50**, 2383–2394 (2013).

[46] Rosendahl, P. L., Staudt, Y., Schneider, A. P., Schneider, J. & Becker, W. Nonlinear elastic finite fracture mechanics: Modeling mixed-mode crack nucleation in structural glazing silicone sealants. *Materials & Design* **182**, 108057 (2019).

[47] Ritter, J., Löwe, H. & Gaume, J. Microstructural controls of anticrack nucleation in highly porous brittle solids. *Scientific Reports* **10**, 12383 (2020).

[48] Shaikeea, A. J. D., Cui, H., O'Masta, M., Zheng, X. R. & Deshpande, V. S. The toughness of mechanical metamaterials. *Nature Materials* **21**, 297–304 (2022).

[49] Hedvard, M. L., Dias, M. A. & Budzik, M. K. Toughening mechanisms and damage propagation in architected-interfaces. *International Journal of Solids and Structures* **288**, 112600 (2024).

[50] McClung, D. M. Shear Fracture Precipated by Strain Softening as a Mechanism of Dry Slab Avalanche Release. *Journal of Geophysical Research* **84**, 3519–3526 (1979).

[51] Heierli, J. & Zaiser, M. An analytical model for fracture nucleation in collapsible stratifications. *Geophysical Research Letters* **33**, L06501 (2006).

[52] Chiaia, B. M., Cornetti, P. & Frigo, B. Triggering of dry snow slab avalanches: stress versus fracture mechanical approach. *Cold Regions Science and Technology* **53**, 170–178 (2008).
